# SD-208, a Novel Protein Kinase D Inhibitor, Blocks Prostate Cancer Cell Proliferation and Tumor Growth *In Vivo* by Inducing G2/M Cell Cycle Arrest

**DOI:** 10.1371/journal.pone.0119346

**Published:** 2015-03-06

**Authors:** Manuj Tandon, Joseph M. Salamoun, Evan J. Carder, Elisa Farber, Shuping Xu, Fan Deng, Hua Tang, Peter Wipf, Q. Jane Wang

**Affiliations:** 1 Department of Pharmacology and Chemical Biology, University of Pittsburgh, Pittsburgh, Pennsylvania, 15261, United States of America; 2 Department of Chemistry, University of Pittsburgh, Pittsburgh, Pennsylvania, 15261, United States of America; 3 Department of Cell Biology, School of Basic Medical Sciences, Southern Medical University, Guangzhou, China; 4 Department of Cellular and Molecular Biology, University of Texas Health Science Center at Tyler, Tyler, Texas, 75708, United States of America; Thomas Jefferson University, UNITED STATES

## Abstract

Protein kinase D (PKD) has been implicated in many aspects of tumorigenesis and progression, and is an emerging molecular target for the development of anticancer therapy. Despite recent advancement in the development of potent and selective PKD small molecule inhibitors, the availability of *in vivo* active PKD inhibitors remains sparse. In this study, we describe the discovery of a novel PKD small molecule inhibitor, SD-208, from a targeted kinase inhibitor library screen, and the synthesis of a series of analogs to probe the structure-activity relationship (SAR) vs. PKD1. SD-208 displayed a narrow SAR profile, was an ATP-competitive pan-PKD inhibitor with low nanomolar potency and was cell active. Targeted inhibition of PKD by SD-208 resulted in potent inhibition of cell proliferation, an effect that could be reversed by overexpressed PKD1 or PKD3. SD-208 also blocked prostate cancer cell survival and invasion, and arrested cells in the G2/M phase of the cell cycle. Mechanistically, SD-208-induced G2/M arrest was accompanied by an increase in levels of p21 in DU145 and PC3 cells as well as elevated phosphorylation of Cdc2 and Cdc25C in DU145 cells. Most importantly, SD-208 given orally for 24 days significantly abrogated the growth of PC3 subcutaneous tumor xenografts in nude mice, which was accompanied by reduced proliferation and increased apoptosis and decreased expression of PKD biomarkers including survivin and Bcl-xL. Our study has identified SD-208 as a novel efficacious PKD small molecule inhibitor, demonstrating the therapeutic potential of targeted inhibition of PKD for prostate cancer treatment.

## Introduction

Prostate cancer is the most common male malignancy in western countries [1] and the second leading cause of cancer death in the US, representing 29% of all male cancer deaths [2]. While localized disease can be treated by a few modalities, the metastatic stage is palliative rather than therapeutic and there are currently no effective therapies. Protein kinase D (PKD) is a family of ubiquitous serine-threonine protein kinase that belongs to the Ca^2+^/ Calmodulin—dependent protein kinase superfamily [3]. The three isoforms of PKD (PKD1/PKCµ[4], PKD2 [5] and PKD3/PKCν [6]) are widely distributed in a variety of tissues, and are homologous in structure and function. PKDs are activated by protein kinase Cs (PKCs) through phosphorylation of two conserved serine residues in the activation loop of the kinase domain. For PKD1, activation involves PKC-mediated phosphorylation at Ser^738^ and Ser^742^ in the activation loop, followed by autophosphorylation at Ser^910^ that conveys full activation [7,8].

PKD plays an important role in mediating mitogenic signaling and has been shown to potentiate the GPCR-induced cell proliferation through the MEK/ERK/RSK pathway [9]. Emerging evidence demonstrates the involvement of PKD in key signaling pathways that regulate tumor cell proliferation such as β-catenin, androgen receptor, mTORC1-S6K1, and MAPK in various tumor cell models [10–15]. Collectively, this mechanistic footprint demonstrates an important role of PKD in cancer, providing the foundation of targeting PKD using small molecule inhibitors for cancer therapy.

In recent years, the development of small molecule inhibitors that target the PKD family has advanced significantly [15–19]. After the discovery of the first potent, selective, and cell-active small molecule inhibitor CID 755673 by our group [20,21] we directed significant efforts at improving its potency and selectivity through chemical modifications. While we developed leads with much improved potency and selectivity, such as kb-NB142-70 [20,22], the *in vivo* efficacy and applicability of this class of inhibitors remained limited [23]. A recent study utilizing targeted libraries of small organic kinase inhibitors has also identified a novel ATP-competitive 4-azaindole scaffold, and a set of pyrazolopyrimidine small molecule inhibitors with low nanomolar potency for *in vitro* inhibition of PKD that potently blocked prostate cancer cells proliferation and growth [18,24,25]. Despite the immense efforts toward the development of potent and selective PKD small molecule inhibitors for cancer therapy, there remains great demand for efficacious *in vivo*-active PKD small molecule inhibitors able to progress into preclinical development and further into the clinical application.

In the present study, we identified SD-208 as a novel ATP-competitive PKD small molecule inhibitor *in vitro* and *in vivo*. In prostate cancer cells, we showed that SD-208 significantly suppressed tumor cell proliferation through targeted inhibition of PKD and blocked tumor cell invasion. A mechanistic investigation suggested that SD-208 abrogated cancer cell proliferation through inducing G2/M cell cycle arrest by increasing cyclin-dependent kinase inhibitor (CDKI)—p21 levels and modulating the activity of Cdc25C/Cdc2 pathway. Further, SD-208 blocked PC3 prostate cancer cell proliferation and the growth of tumor xenografts in nude mice, correlating to decreased Bcl-xL and survivin. We also generated a series of analogs of SD-208 by chemical synthesis, and evaluated their inhibitory profile, demonstrating a narrow SAR for this lead structure. Our results thus characterize SD-208 as a structurally new PKD small molecule inhibitor that significantly abrogates prostate cancer cell proliferation *in vitro* and *in vivo*.

## Materials and Methods

### Ethics Statement

This study was carried out according to Institutional Animal Care and Use Committee (IACUC) at the University of Pittsburgh guidelines and the animal protocol and study was approved by University of Pittsburgh Institutional Animal Care and User Committee (Protocol Number: 11120102).

### Cell culture and reagents

Human prostate carcinoma PC3, DU145 and LNCaP cells were obtained from American Type Culture Collection (ATCC) and cultured according to the manufacturer’s recommendation. All cell lines were grown at 37°C, 5% CO_2_ in a humidified atmosphere. Ham’s F-12 and MEM were from Cellgro (Manassas, VA) and other culture materials were from Invitrogen (Life Technologies, Carlsbad, CA). Kinase active recombinant GST-tagged human protein kinase D1 (PKD1) was obtained from Enzo Life Sciences (Farmingdale, NY). DMSO was purchased from Sigma (Sigma-Aldrich, St. Louis, MO). Recombinant PKCα, PKCδ, and CAMKIIα were obtained from SignalChem (Richmond, BC, Canada). ATP was purchased from Fisher Scientific (Fair Lawn, NJ). HDAC5 substrate peptide was synthesized by Biobasic Canada Inc. (Markham, ON). Myelin basic protein 4–14 was purchased from AnaSpec Inc. (Fremont, CA). A pharmacologically active kinase inhibitor library was purchased from Tocris Bioscience (Minneapolis, MN).

### Synthesis of SD-208 Analogs

We decided to chemically modify the 5-chloro-2-fluorophenyl (*zone 1*) and the *para*-aminopyridine (*zone 2*) moieties of SD-208 to probe the contributions of these side chains to PKD1 inhibition (Table 1 and S1 Fig.). Details of synthesis are described in the *Supporting Information*.

### Docking of SD-208

This study was conducted as previously reported [25]. The structural homology model of PKD1’s kinase domain (residues 587 to 835) was generated using the I-TASSER server. Sybyl-X 2.1 Surflex-Dock software performed the docking of SD-208 into PKD1’s kinase domain. Program settings were configured under the subsequent parameters. All hydrogen atoms were present in both the homology model as well as SD-208. The docking area was restricted within the confines of the ATP binding site, which include the following residues: Ala^610^, Lys^612^, Met^659^, Glu^660^, Lys^661^, Leu^662^, His^663^, Glu^710^, Leu^713^, and Cys^726^. Twenty additional starting conformations were evaluated for SD-208 and all binding modes were assessed. Molecular modeling was presented using PyMol.

### *In Vitro* Radiometric PKD Inhibitor Screening Assay

An *in vitro* radiometric PKD kinase assay was used to screen a small library of 80 commercially available kinase inhibitors, Tocriscreen kinase inhibitor collection (Tocris Biosciences, Minneapolis, MN), for PKD1 inhibitory activity at 1 µM concentration [24]. *In Vitro* Radiometric protocol is described in the *Supporting Information*.

### Western Blot Analysis

LNCaP and DU145 prostate cancer cells were maintained in RPMI 1640, while PC3 cells were maintained in Ham’s F-12 medium, supplemented with 10% fetal bovine serum (FBS) and 1000 units/L penicillin, and 1 mg/mL streptomycin in 5% CO_2_ at 37°C. A Western blot analysis was carried out as previously reported [10]. Primary antibodies targeted p-S^916^-PKD1 (human p-S^910^-PKD1) (Millipore), p-S^744/748^-PKD1 (human p-S^738/742^-PKD1), PKD1, pHsp 27, Hsp 27,p21, Cyclin A2, p-Cdc2, Cdc2, Cyclin B1, p-Cdc25C, Cdc25C, Survivin, Bcl-XL, Akt and p-S^473^-Akt (Cell Signaling Technology), PKD2 (Abcam), Cyclin D1 (Santa Cruz Biotechnology, Dallas, TX), GAPDH (Enzo Life Science, Farmingdale, NY) and tubulin (Sigma) were used for blotting.

### In Vitro Radiometric PKC and CAMKIIα Kinase Assay

The PKC kinase assay was carried out by co-incubating 1 µCi [γ-^32^P]ATP, 20 µM ATP, 50 ng of purified PKCα or PKCδ and 5 µg of myelin basic protein 4–14, 0.25 mg/mL bovine serum albumin, 0.1 mg/mL phosphatidylcholine/phosphatidylserine (80/20%) (1 µM), 1 µM phorbol dibutyrate in 50 µL of kinase buffer containing 50 mM Tris-HCl, pH 7.5, 4 mM MgCl_2_ and 10 mM β-mercaptoethanol. For the CAMK assay, 50 ng of CAMKIIα and 2 µg syntide-2 substrate in 50 µL kinase buffer were incubated with 0.1 mM MgCl_2_, 1 µCi of [γ-^32^P] ATP, 70 µM ATP. 0.5 mM CaCl_2_ and 30 ng/µL calmodulin were preincubated for 15 min on ice and then added in the kinase reaction. The reactions were incubated at 30°C for 10 min and 25 µL of the reaction was spotted on Whatman P81 filter paper. The filter paper was washed 3 times in 0.5% phosphoric acid, air dried and counted using Beckman LS6500 multipurpose scintillation counter.

### MTT Assay

PC3 cells were seeded into 96-well plates (3000 cells/well) and allowed to attach overnight. Cells were then incubated in media containing 0.7–100 μM inhibitors for 72 h. 50 μL of 3-(4,5-Dimethylthiazol-2-yl)-2,5-diphenyltetrazolium bromide methyl thiazolyl tetrazolium (MTT) solution at 2 mg/mL concentration was added to each well and incubated for 4 h at 37°C. Then, media was removed and 200 μL DMSO was added to each well. The plate was mixed by shaking for 5 min and the optical density was determined at 570 nm.

### Subcutaneous Prostate Tumor Xenograft Study

Athymic NCr-nu/nu mice (4–6 week old) (NCI, Frederick, MD) were injected subcutaneously with 1.4 X 10^6^ PC3 cells in 100 µL media. Three days after inoculation, when tumors became palpable, the mice were randomized into the following groups (5 mice per group); (a) vehicle (control) 1% (w/v) methylcellulose administered by oral gavage twice daily; (b) SD-208 60 mg/kg suspended in 1% methylcellulose administered by oral gavage twice daily, for 21 days. Body weights were measured twice weekly. Tumors were measured in two dimensions every 2 to 3 days by calipers and tumor volume was calculated as *V* (mm^3^) = (*L X W* ^2^)/2. The tumor volumes were compared at each time point among groups using unpaired t test and a *p* value of < 0.05 was considered statistically significant. Twenty-four days after inoculation, mice were euthanized by CO_2_ inhalation and tumors were excised. All animal studies were conducted in accordance with an Institutional Animal Care and Use Committee (IACUC) at the University of Pittsburgh.

### Immunohistochemical (IHC) Staining of Tumor Tissues

Formalin-fixed and paraffin embedded sections were stained as described earlier [10]. Briefly, sections were deparraffinized by xylene and rehydrated in reducing gradients of ethanol. Antigen retrieval was performed by simmering the slides at near boiling temperature for 30 min in 10 nmol/L sodium citrate buffer (pH 6.0), followed by cooling to room temperature. Tissue sections were then stained with Ki-67 (Genetex, Irvine, CA) and cleaved caspase-3 (Cell signaling Technology, Beverly, MA) antibodies at 4°C overnight and then incubated with biotinylated goat anti-rabbit antibody (Vector laboratories, Logan, UT). The slides were then developed using Vectastain ABC Standard kit and AEC Reagent (Scytek Labs,Logan, UT). The tissues were finally counterstained with hematoxylin. A total of 10 fields were examined and counted from each treatment groups in a blinded fashion.

### Immunoprecipitation and p-PKD2 Kinase Assay

PKD2 was immunoprecipitated by incubating 500 μg of total cell lysates from tumor explants with protein A/G Sepharose beads (Santa Cruz Biotechnology, Inc.) preconjugated with anti-PKD2 antibody (GeneTex, Irvine, CA.) overnight at 4°C with constant rotation. The immunocomplexes were then pelleted and washed with washing buffer (50 mM Tris-HCl, pH 7.4, 150 mM sodium chloride, and 1% Triton X-100). Equal amounts of immunocomplex were then subjected to PKD kinase assay, as described in supplement protocol. In brief, the assay was carried out by coincubating 20 μl of immunoprecipitated PKD2 with 25 μM ATP, 0.2 μl of [γ-^32^P]ATP (PerkinElmer Life and Analytical Sciences, Boston, MA) and 1.2 μM a 25 a.a. HDAC-5 peptide as substrate [26] in a final volume of 50 μl. The reaction was allowed to proceed at 30°C for 10 min. An aliquot of the reaction mixture was then spotted on p81 paper, washed in 5% phosphoric acid and counted.

### Cell Proliferation Assay and Cell Cycle Analysis

Proliferation of PC3 cells was measured by counting the number of viable cells upon trypan blue staining as previously described [10]. Cell cycle analysis was performed as described [22]. Briefly, PC3 cells were treated with the appropriate compounds at 30 μM for 72 h, and then fixed in 70% ice-cold ethanol overnight, followed by labeling with propidium iodide. The labeled cells were analyzed using a FACSCalibur flow cytometer (BD Biosciences, San Jose, CA).

### Matrigel Invasion Assays

Matrigel invasion assay was conducted as previously described [21,22] (see *Supporting Information* for more details).

### Statistical analysis

Statistical analysis was completed using GraphPad Prism software 5.0. A *p* value of < 0.05 was considered statistically significant.

## Results

### Identification of SD-208 as a novel PKD small molecule inhibitor

**Fig 1:**
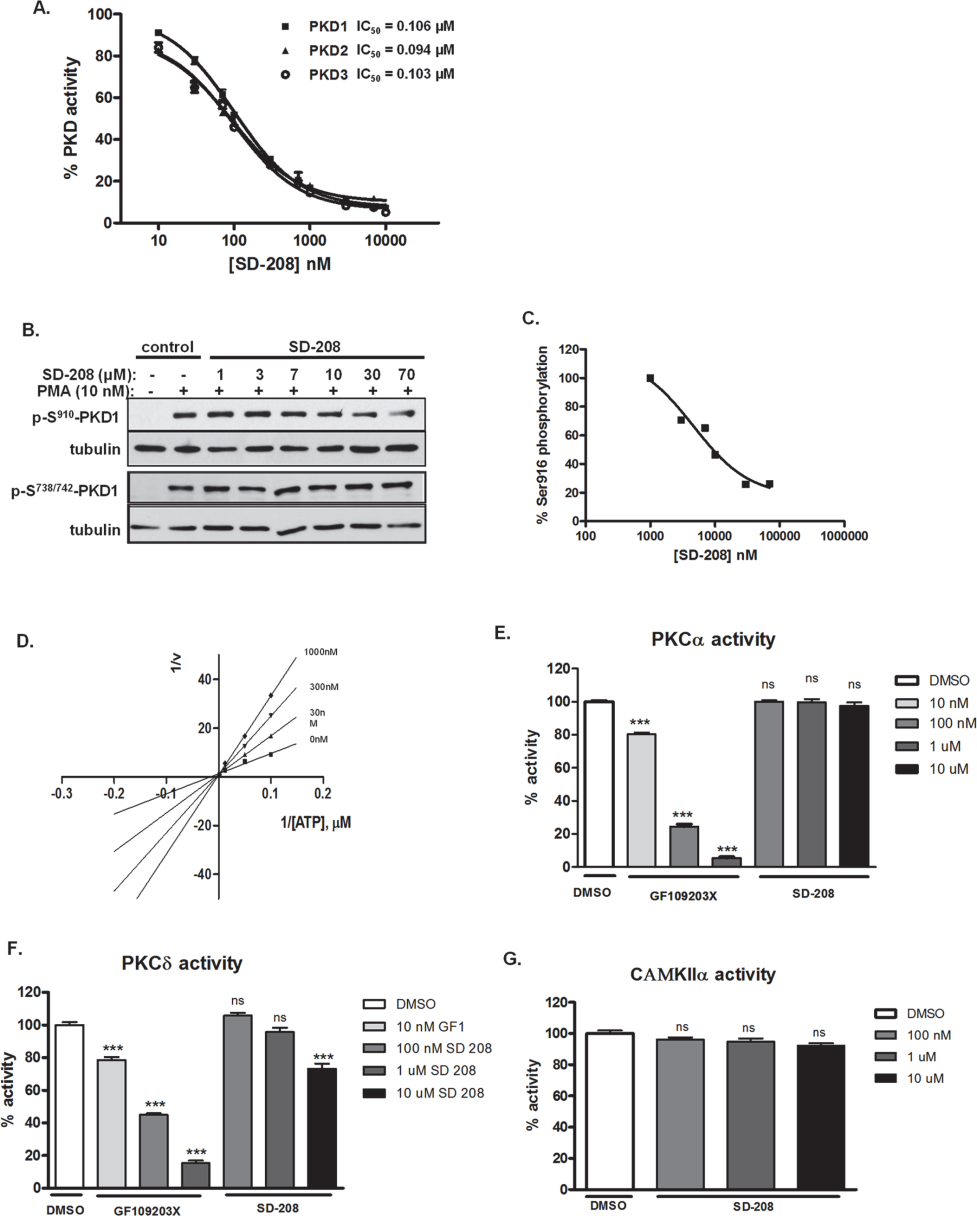
SD-208 was a cell-active, ATP-competitive PKD inhibitor and did not inhibit PKC and CAMK. **A. Determination of PKD kinase activity *in vitro***. Inhibition of recombinant human PKD1, 2 and 3 was assayed in the presence of 10 different concentrations of SD-208 by an *in vitro* radiometric PKD kinase assay. The IC_50_ values were calculated as the mean ± SEM of at least three independent experiments with triplicate determinations at each compound concentration in each experiment. The data were plotted as a function of inhibitor concentration and a representative graph is shown. **B**. **SD-208 inhibited PMA-induced PKD1 activation in prostate cancer cells**. LNCaP cells were pretreated with different doses of inhibitors for 45 min, followed by PMA stimulation at 10 nM for 20 min. Cell lysates were subjected to immunoblotting for p-S^910^-PKD1 and p-S^738/742^-PKD1. Tubulin was blotted as loading control. The experiment was repeated three times and the representative blots are shown. **C. Determination of cellular IC**_**50**_ ****. Western blots from ‘B” were quantified using densitometry analysis. The data were plotted and IC_50_ values were derived from the concentration-response curves using GraphPad. One of the three concentration-response curves is shown. **D. SD-208 is an ATP-competitive kinase inhibitor**. PKD1 kinase activity was measured as a function of increasing concentrations of ATP in the presence of varying concentrations of SD-208. Lineweaver-Burke plots of the data are shown. Data presented were the mean ± S.E. of three independent experiments with triplicate determinations at each data point in each experiment. **E-F. Selectivity of SD-208 against related kinases**. Inhibition of PKCα (**B**) or PKCδ (**C**) was determined at 10 nM, 100 nM, 1μM, and 10 μM. As controls, the PKC inhibitor GF109203X potently inhibited PKCα and PKCδ activity. Data are the mean ± SEM of two independent experiments. **G**. Inhibition of CAMKIIα was measured by the radiometric CAMK kinase assay. Data are the mean ± S.E. of two independent experiments with triplicate determinations at each data point in each experiment. Statistical significance was determined using the unpaired t-test. ns, not significantly significant; *, p<0.05; **, p<0.01; ***, p<0.001.

We conducted a screen of PKD small molecule inhibitors on Tocriscreen kinase inhibitor collection of 80 chemically diverse kinase inhibitors at 1 µM concentration using an *in vitro* radiometric PKD kinase assay. With a cut-off set at ≥ 50% inhibition of total PKD1 kinase activity, 16 compounds were identified as primary hits. Among them, SD-208 suppressed 82% of PKD1 activity at 1 μM concentration (data not shown) and was further evaluated for inhibition of PKD1, 2 and 3 in a 10-point concentration curve using the radiometric PKD kinase assay [21]. As shown in **Fig. 1A**, SD-208 inhibited PKD1, 2, and 3 with an IC_50_ of 106.87 ± 6.6 nM (n = 9), 93.54 ± 2.7 nM (n = 9), and 105.3 ± 2.6 nM (n = 9), respectively. Using LNCaP prostate cancer cells as the model system, the effect of SD-208 on 12-myristate 13-acetate (PMA)-induced endogenous PKD1 activation was examined as previously described [10,22]. As shown in **Fig. 1B-C**, treatment of LNCaP cells with 10 nM of PMA for 20 min induced robust PKD1 activation, detected by increased PKD1 activation loop phosphorylation at Ser^738/742^ conferred by PKC and C-terminal autophosphorylation at Ser^910^. Pretreatment with increasing concentrations of SD-208 concentration-dependently inhibited autophosphorylation at Ser^910^ of PKD1 with an IC_50_ of 17.0 ± 1.5 μM (**Fig. 1C**). In contrast, PKC-dependent trans-phosphorylation at Ser^738/742^ was unaffected by SD-208, indicating that SD-208 directly abrogated PKD1 catalytic activity without blocking upstream PKC-mediated PKD1 trans-phosphorylation. Thus, SD-208 is structurally distinct pan-PKD inhibitor that elicits targeted inhibition of PKD1 kinase activity in prostate cancer cells.

### SD-208 was an ATP-competitive inhibitor with high selectivity for PKD over closely related kinases

To gain a better insight on the mode of action for SD-208, we examined the effects of increasing concentrations of ATP on PKD1 inhibition. Lineweaver-Burk plots were generated by plotting the reciprocal of reaction velocities (1/v) against the reciprocal of ATP concentrations (1/[ATP]) at different compound concentrations. The points were fitted by linear regression. As shown **Fig. 1D**, all lines converged on the Y-axis, indicating that SD-208 was an ATP-competitive inhibitor. Next, we determined the specificity of SD-208 for PKD by examining their activity for the classical and novel PKC isoforms using PKCα and PKCδ. No significant inhibitory activities for PKC isoforms were detected at 0.1 and 1 μM and for PKCα at 10 μM (**Fig. 1E-F**). The potent PKC inhibitor GF109203X was tested as a positive control and it potently and concentration-dependently inhibited PKCα and PKCδ. High sequence homology of PKD with the CAMK family led us to investigate the inhibition of CAMKIIα by SD-208. As illustrated in **Fig. 1G**, SD-208 showed no activity on CAMKIIα up to 10 μM. Taken together, these data indicate that SD-208 is a highly specific inhibitor for PKD relative to other closely related kinases including PKCs and CAMKs.

### Synthesis, SAR analysis, and modeling of SD-208 and its analogs

**Fig 2:**
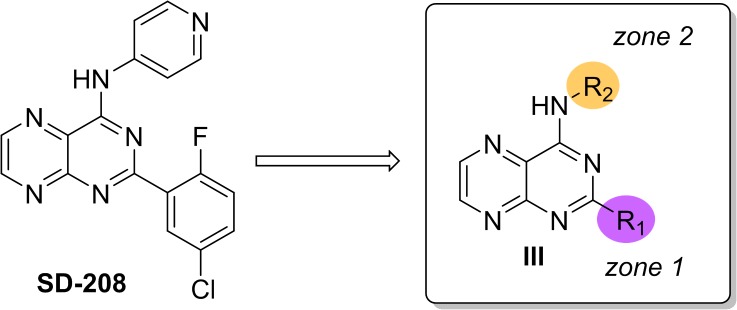
Scope of first-generation SAR studies on SD-208. 2-Zone model for SD-208 analog synthesis.

Our approach to the synthetic studies, as detailed in **S1 Fig.**, was based on two zones of substitution in SD-208, where the 2-fluoro-5-chlorobenzene represents zone 1 and 4-aminopyridine represents zone 2 (**Fig. 2**). Using the pteridine core as a synthetic platform, we were interested in exploring the substitutions attached to the fused pyrimidine ring, specifically to identify the SAR contributions of the fluorine and chlorine substituents in zone 1 and the pyridine ring in zone 2. The secondary amine in zone 2 was preserved because amino substitutions alpha to nitrogen atoms in heterocycles are often implicated in protein kinase hinge binding [27,28]. As a consequence, the secondary amine may prove essential for activity.

Our SAR investigation initially focused on the importance of the substituents on the aromatic ring in zone 1 (**Table 1**). The electron density of the aromatic ring in SD-208 is decreased by the electronegativity of the fluorine atom [29,30]. Moreover, chlorine may contribute to dipole-dipole interactions in the protein binding cleft [31]. Removal of the halogens from the benzene ring (**5a-d**) led to a significantly decreased activity. In contrast, installation of electron withdrawing groups such as fluorine (**5e-g**) and trifluoromethyl (**5h**) restored activity, but only when zone 2 was comprised of 4-aminopyridine (**5e,h**). Interestingly, only 4-aminopyridine was tolerated in zone 2. Therefore, our attempts to improve the solubility with piperazine (**5j**) and 2-morpholinoethyl (**5g**) substituents failed to conserve PKD1 inhibitory effects. Surprisingly, even a regioisomer, 3-aminopyridine (**5d,f**), was not tolerated. This observation suggests that the nitrogen in the pyridine ring may be involved in essential hydrogen bonding interactions, but only in a specific spatial arrangement. Additional diversification of zone 1 with 3,5-dichloro substituents (**5i**) did not restore activity. This further supports the importance of the electron-withdrawing group since chlorine is less electronegative than fluorine. Changing the positions of substituents on the benzene ring in the 2,5-difluoro- (**5e**) and 3-trifluoromethyl- (**5h**) analogs conserved activity. This result indicates that the position of the substituents on this arene may not be critical as long as the functional group is sufficiently electron-withdrawing.

**Table 1:** Overview of structures and yields of intermediates 3 and 4 (shown in “S1 Fig.”) and analogs 5 (as described in “***Supporting Information***”) with PKD1 inhibition data for analogs 5.

Entry	Compound	R_1_	R_2_	Yields 3 (%)	Yields 4 (%)	Yields 5 (%)	% PKD1 Inhibition
1	SD-208	(2-F-5-Cl)Ph	4-Pyridyl	78	86	40^b^	74%
2	**5a**	Ph	Ph	72	74	40^c^	≤ 10
3	**5b**	Ph	*c*-Hexyl	72	74	21^c^	≤ 10
4	**5c**	Ph	4-Pyridyl	72	74	71^b^	≤ 10
5	**5d**	Ph	3-Pyridyl	72	74	65^b^	≤ 10
6	**5e**	(2,5-F_2_)Ph	4-Pyridyl	57	50	<5^b^	60
7	**5f**	(2,5-F_2_)Ph	3-Pyridyl	57	50	51^b^	≤ 10
8	**5g**	(2,5-F_2_)Ph	2- Morpholinoethyl	57	50	52^b^	≤ 10
9	**5h**	(3-CF_3_)Ph	4-Pyridyl	56	53	23^b^	60
10	**5i**	(3,5-Cl_2_)Ph	4-Pyridyl	61	54	<5^b^	≤ 10
11	**5j**	(2-F-5-Cl)Ph	Piperazinyl^a^	78	86	13^b^	≤ 10

^a^Piperazine is directly connected to the pteridine core without a secondary amine.

^b^Via the PyBOP route.

^c^Via the chlorination/substitution route (2-step yield).

**Fig 3:**
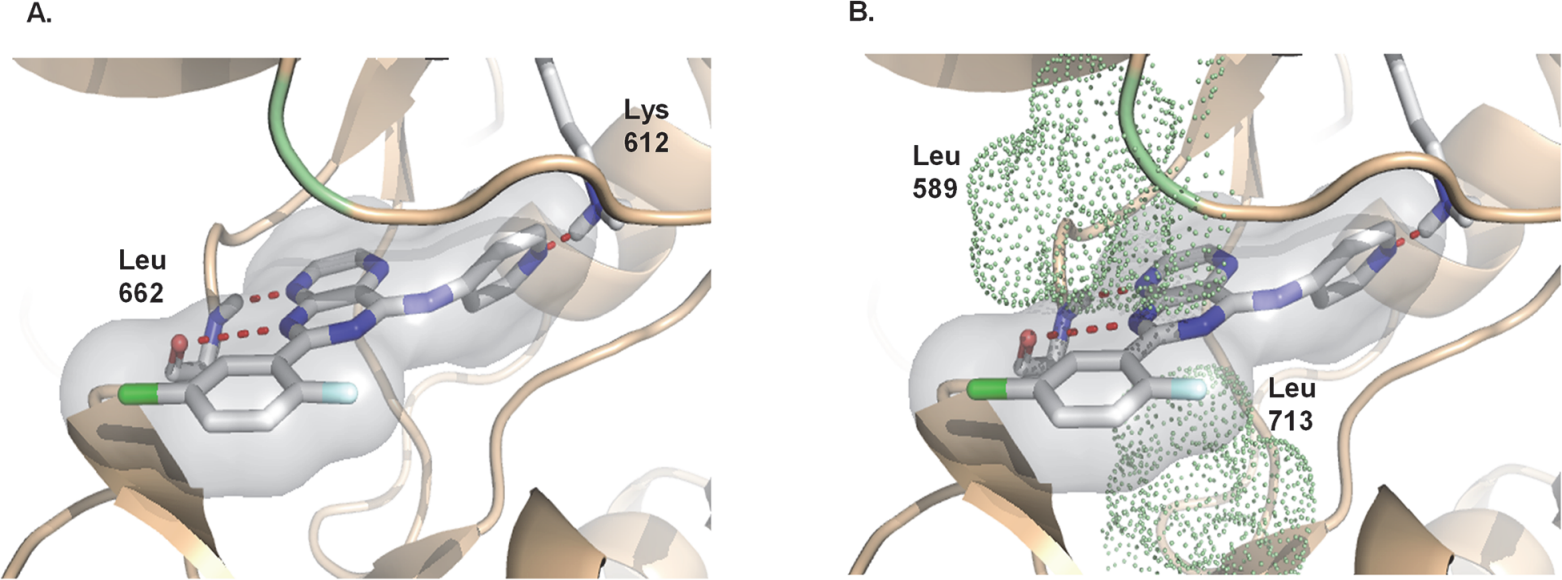
Molecular modeling of SD-208 in the active site of a PKD1 homology model. This depiction demonstrated a potential binding mode of SD208 in the active site of PKD1. Red dotted lines represent key interactions between SD-208 and PKD1.

In conjunction with this SAR analysis, we employed our PKD1 homology model [25] to rationally explore a putative binding mode of SD-208 within the active site of PKD1. Common among ATP-competitive inhibitors, SD-208 was shown in **Fig. 3A** to interact with the hinge region through hydrogen bond formation between the amino acid backbone of Leu^662^ and its pteridine core. Additionally, a potentially critical hydrogen bond was observed with the nitrogen from the 4-aminopyridine and the charged side chain of Lys^612^. A similar interaction was also reported in other known PKD1 inhibitors [18]. Our SAR analysis further supports the notion that the nitrogen from the 4-aminopyridine acts as a valuable hydrogen bond acceptor. Ablation of this bond revealed a marked decrease in PKD1 inhibition. Moreover, displayed in **Fig. 3B**, our model positions the disubstituted phenyl ring in a hydrophobic pocket consisting of Leu^589^ and Leu^713^, which may dictate the tolerance of aromatic substitutions. Although other binding modes of SD-208 were explored, the current model best recapitulated results represented in our SAR analysis.

### SD-208 inhibited prostate cancer cell proliferation and invasion

**Fig 4:**
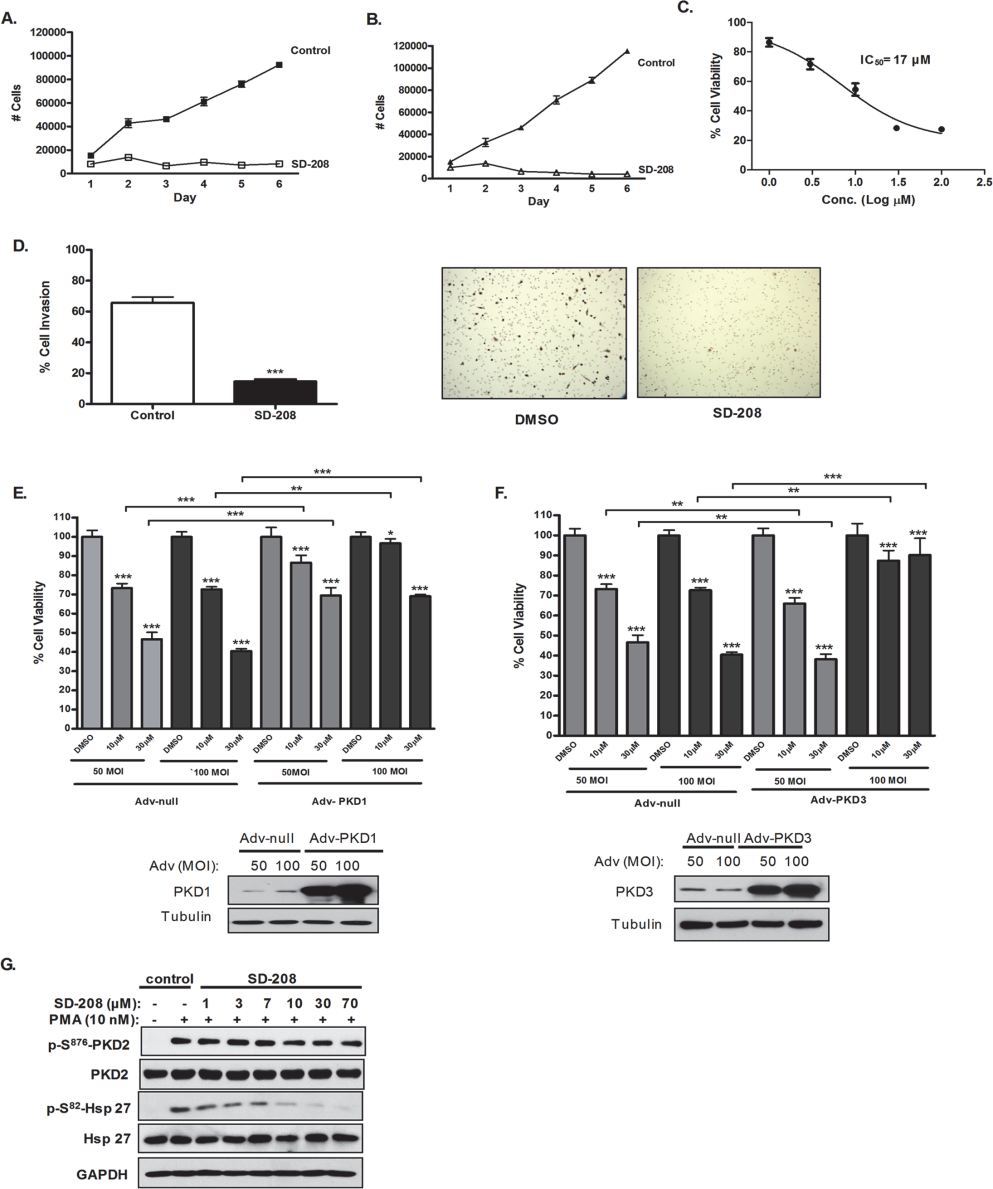
SD-208 inhibited prostate cancer cells proliferation, survival, and invasion and the anti-proliferative effect of SD-208 was mediated through the inhibition of PKD. **A-B. SD-208 inhibited PC3 (A) and LNCaP (B) prostate cancer cell proliferation**. PC3 and LNCaP cells were plated in triplicates in 24-well plates. Cells were allowed to attach overnight. A cell count at day 1 was made, and then either a vehicle (DMSO) or SD-208 at 30 μM was added. Cells were counted daily for a total of 6 days. Data are the mean ± S.E. of two independent experiments with triplicate determinations at each data point in each experiment. **C**. **SD-208 inhibited PC3 prostate cancer cell survival**. PC3 cells were seeded into 96-well plates (3000 cells/well) and were then incubated in media containing 0.3–100 μM inhibitors for 72 h. MTT solution was added to each well and incubated for 4 h. Optical density was read at 570 nm to determine cell viability. The IC_50_ was determined as the mean of three independent experiments for each compound. **D. SD-208 inhibited prostate cancer cell invasion**. DU145 cells were incubated with 30 μM SD 208 in Matrigel inserts. After 20 h, noninvasive cells were removed and invasive cells were fixed in 100% methanol, stained in 0.4% hematoxylin solution, and photographed. The number of cells that invaded the Matrigel matrix was determined by cell counts in 6 fields relative to the number of cells that migrated through the control insert. Percentage invasion was calculated as the percent of the cells invaded through Matrigel inserts vs. the total cells migrated through the control inserts. Data are the mean ± S.E. of three independent experiments with triplicate determinations at each data point in each experiment. Statistical significance was determined using the unpaired t-test. ***, p<0.001. **E-F. Overexpression of PKD1 and PKD3 in prostate cancer cells rescued the anti-proliferative effects of SD-208**. PC3 (0.5 million) cells were seeded in a 60 mm dish and infected the next day with 50 and 100 MOI of PKD1 and PKD3 adenoviruses (Adv-PKD1 and Adv-PKD3). Empty adenovirus (Adv-null) was used as control. After 24 h, 3000 cells/well were plated in 96-well plates and treated with and without 10 and 30 μM SD-208 for 72 h. MTT solution was added to each well and incubated for 4 h. Optical density was read at 570 nm to determine cell viability. The overexpression of PKD1 and PKD3 was confirmed by Western blotting analysis. This experiment was repeated three times and data are the mean ± S.E. of all three independent experiments. Statistical significance between DMSO and inhibitor treatment was determined using the unpaired t-test.*, p<0.05; **, p<0.01; ***, p<0.001. **G. PKD mediated Hsp27 activity in prostate cancer cells was inhibited by SD-208**. DU145 cells were pretreated with different doses of inhibitors for 45 min, followed by PMA stimulation at 10 nM for 20 min. Cell lysates were subjected to immunoblotting for p-S^910^-PKD1 and p-S^738/742^-PKD1. GAPDH was blotted as loading control. The experiment was repeated two times and the representative blots are shown.

We next investigated the effects of targeted inhibition of PKD by SD-208 on prostate cancer cell proliferation, survival, and cell cycle progression. As shown in **Fig. 4A-B**, SD-208 at 30 μM caused significant reduction in PC3 prostate cancer cells proliferation starting at day 2 and persisted to the end of the experiment. SD-208 also concentration-dependently induced cell death with an IC_50_ of 17.0 ± 5.7 μM (n = 3) (**Fig. 4C**). The effect of SD-208 on tumor cell invasion was assessed using Matrigel invasion assay (cell invasion). As illustrated in **Fig. 4D**, treatment of cells with 30 μM SD-208 for 20 h resulted in over 60% inhibition of cell invasion compared with control, indicating that SD-208 significantly blocked tumor cell invasion. Taken together, our data demonstrates that SD-208 is a potent inhibitor of prostate cancer cell proliferation and invasion.

### SD-208-induced growth arrest was mediated through targeted inhibition of PKD

To determine the target specificity of SD-208 at the cellular level, we sought to investigate if SD-208-induced anti-proliferative effects were mediated though targeted inhibition of PKD. An attempt was made to reverse the effect of the PKD inhibitor via overexpressing different PKD isoforms. As shown in **Fig. 4E-F**, PC3 cells were infected with null adenovirus (Adv-null) and adenovirus carrying PKD1 and PKD3 genes (Adv-PKD1 and Adv-PKD3) at 50 and 100 MOI. Western blotting revealed overexpression of PKD1 and PKD3 in PC3 cells infected with adenoviruses carrying the respective genes. The infected cells were subjected to SD-208 treatment at 10 and 30 μM. Expression of increasing levels of PKD1 and PKD3 reversed the anti-proliferative effects of SD-208. The higher levels of PKD1 or PKD3 expression, the greater the rescue effects, and at 100 MOI a nearly complete reversal of inhibition of cell proliferation was observed for Adv-PKD3 at 10 and 30 µM SD-208 (**Fig. 4**). These data indicate that the anti-proliferative effects of SD-208 are mediated through the inhibition of PKD. In order to determine whether anti-proliferative property of SD-208 is due to targeted inhibition of PKD, we seek to determine if it also inhibited PKD mediated PMA-induced Hsp27 phosphorylation at Ser-82 in prostate cancer cells (Yuan and Rozengurt, 2008). Hsp27 is a well established direct substrate of PKD [32,33]. Cultures of DU145 cells were treated in the absence or in the presence of increasing concentration of SD-208 for 45 min and then stimulated with 100 nM PMA. As indicated in **Fig. 4G** treatment of prostate cancer cells with PMA for 20 min induced robust PKD1 and Hsp27 activation, detected by increased PKD1 autophosphorylation at Ser^910^ and Hsp27 phosphorylaion at ^Ser82^. However pretreatment with increasing concentrations of SD-208 concentration-dependently inhibited PKD induced activation of Hsp27 at Serine 82 along with PKD1 and PKD2 phosphorylation at Serine 910 and Serine 876, respectively. Similar results were also observed when LnCAP and PC3 cells lines were treated with SD-20 (**S2 and S3 Figs**.). Our data, for the first time, demonstrates the specificity the inhibitory effects of SD-208 on PKD and its direct target Hsp27.

### SD-208 caused G2/M cell cycle arrest by inducing Cip1/p21 and upregulating Cdc25C/Cdc2 pathway activity

**Fig 5:**
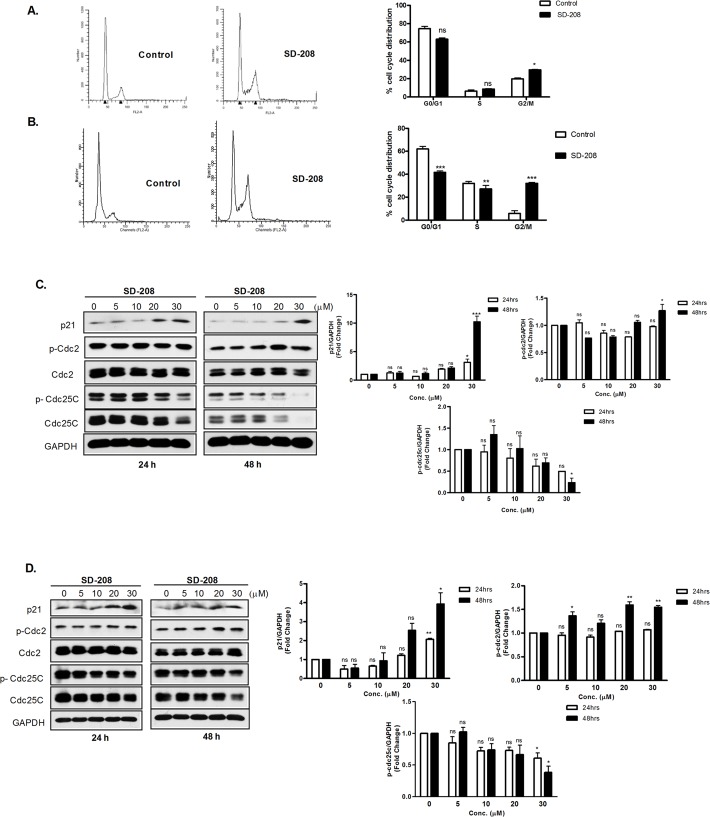
SD-208 arrested cells in G2/M and regulated the levels and activities of cell cycle regulatory proteins at the G2/M phase of cell cycle in prostate cancer cells. **A-B. SD-208 induced G2/M cell cycle arrest in prostate cancer cells**. DU145 cells **(A)** and PC3 cells **(B)** were treated with either vehicle (DMSO) or 30 μM SD 208 for 48 h. Cell cycle distribution was determined by flow cytometry after propidium iodide labeling of fixed cells. The cell cycle plots are representative of three independent experiments (*left*). Data in the bar graph are the mean ± SEM of three independent experiments (*right*). Statistical significance was determined using the unpaired t-test and is indicated. **, p<0.01; ***, p<0.001. **C-D. Effects of SD-208 on the expression and activities of G2/M cell cycle regulatory proteins**. DU145 (**C**) and PC3 (**D**) cells were treated with DMSO or 5–30 µM SD-208 for 24 and 48 h. At the end of each treatment, cells were harvested and subjected to immunoblotting for the cell cycle regulatory proteins as indicated. GAPDH was blotted as loading control. The densitometry data (mean ± SEM from four experiments) were plotted as ‘fold change’ over the control after normalization with GAPDH. The experiments were repeated four times and representative blots from one experiment are shown.

To gain insights in SD-208-induced growth inhibition, we determined the effect of SD-208 on cell cycle progression by flow cytometry. As shown in **Fig. 5A-B**, cell cycle analysis was conducted after treating DU145 and PC3 cells with 30 μM of SD-208 for 72 h. SD-208 hampered cell cycle progression by arresting the cells in G2/M phase of the cell cycle. Specifically, SD-208 significantly increased the proportion of cells in G2/M phase of the cell cycle from 18.78% (vehicle treated cells) to 30.09% in DU145 cells and 6.3% (vehicle treated cells) to 35.6% (30 µM SD-208 treated cells) in PC3 cells, implying that the growth inhibition caused by SD-208 in prostate cancer cells is a consequence of an arrest of cells in G2/M phase of cell cycle.

Cell cycle G2/M transition is positively regulated by CDK 1 (Cdc2) and Cyclin B complex. Cdc25 family of phosphatases plays a crucial role in cell cycle regulation through dephosphorylation of the inhibitory phosphorylations on Cdc2 at Thr^14^ and Tyr ^15^, caused by Wee1 [34]. Moreover, cyclin dependent kinase inhibitor (CDKI), Cip1/p21, also regulates the transitions of G2/M phase by binding and inhibiting the activity of Cdc2-Cyclin B1 complex [35,36]. To determine the mechanisms through which SD-208 induced G2/M arrest in prostate cancer cells, we next assessed the impact of SD-208 on cell cycle machineries at the G2/M transition in DU145 and PC3 prostate cancer cells, which have similar PKD isoform expression patterns [10] As shown in **Fig. 5C-D**, treatment with increasing concentrations of SD-208 for 24 and 48 h induced the expression of Cip1/p21 in a concentration-dependent manner. Additionally, in both the cell lines, SD-208 treatment for 24 and 48 h caused a concentration-dependent decrease in Cdc25C and p-Cdc25C levels, accompanied by a parallel increase in p-Cdc2. These findings suggest that the induction of Cip1/p21, together with the decreased Cdc25C protein and consequently increased phosphorylation of Cdc2 and inhibition of Cdc2/cyclin B1, are the main mechanisms that accounts for the induction of G2/M phase cell cycle arrest by SD-208.

### SD-208 inhibited the growth of tumors in a subcutaneous prostate cancer xenograft model

**Fig 6:**
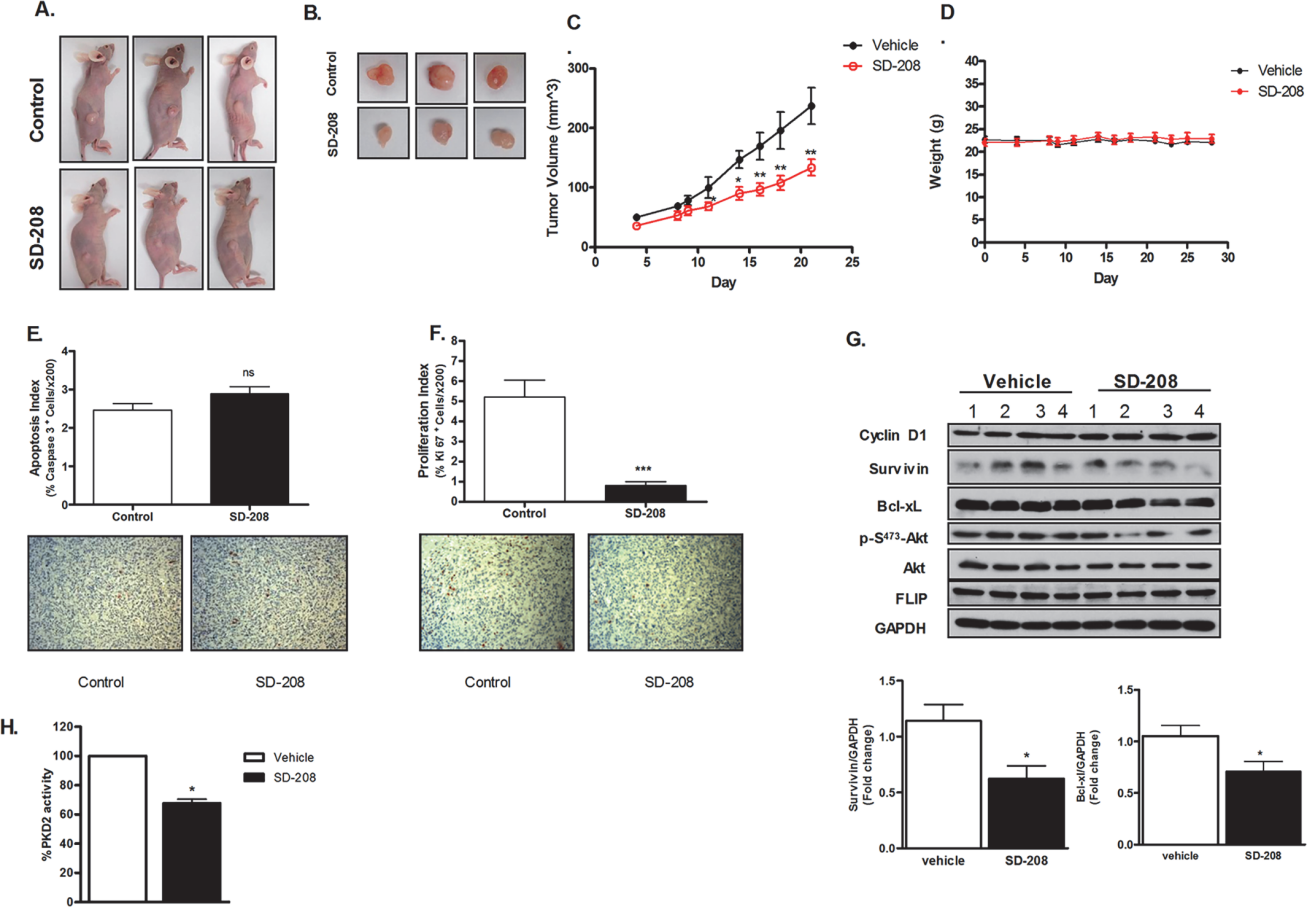
SD-208 inhibited the growth of PC3 tumor xenografts *in vivo*. **A-D. Effects of SD-208 on the growth of PC3 tumor xenograft**. PC3 cells (1.4 X 10^6^) were injected s.c. to both flanks of nude mice. Once tumors were palpable, mice were randomized into two groups (5 mice/group) and were administered 0.2 ml twice daily doses of vehicle [1% (w/v) methylcellulose] or SD-208 (60 mg/kg) by oral gavage. Tumor volume (**C**) and mouse weight (**D**) were measured every 2 to 3 days. Twenty-four days after treatment, mice were euthanized by CO_2_ inhalation, and tumors were excised. Images of representative mice (**A**) and dissected tumors (**B**) are shown. All animal studies were conducted in accordance with the Institutional Animal Care and Use Committee (IACUC). Statistical significance was determined using the unpaired t-test. **, p<0.01; ***, p<0.001. **E**, **Analysis of apoptotic marker cleaved caspase-3 by IHC**. Quantification of *in situ* cleaved caspase-3-positive cells as described in *Materials and Methods*. Representative images of cleaved caspase-3 staining (x200) are shown. **F**, **Analysis of proliferative marker Ki67 by IHC**. Quantification of Ki-67-positive cells or proliferation index as described in *Materials and Methods*. Representative images of Ki-67 staining (x200) are shown. **G. Analysis of PKD-regulated biomarkers in PC3 tumor xenografts**. Cell lysates were prepared from tumor explants obtained from vehicle and SD-208-treated mice. Levels of PKD-regulated proteins were analyzed by Western blotting analysis. The densitometry data represents ‘fold change’ as compared with vehicle control after normalization with GAPDH as loading control. **H. Effect of SD-208 on the PKD2 activity in PC3 tumor xenografts**. Cell lysates from tumor explants obtained from vehicle and SD-208-treated mice were immunoprecipitated with PKD2 antibody followed by *in vitro* kinase assay as described in Material and Methods. The experiment was repeated three times and data are the mean ± S.E. of all three independent experiments with triplicate determinations at each data point in each experiment.

Athymic NCr-nu/nu mice (4–6 weeks old) were inoculated subcutaneously with 1.4 X 10^6^ PC3 cells. Once tumors are palpable (approximately four days after inoculation), mice were randomized into 2 groups of 5 mice and administered orally with vehicle or 60 mg/kg of SD-208. As shown in **Fig. 6A-C**, oral administration of SD-208 for 21 days showed a time-dependent inhibition of tumor growth. A statistically significant reduction in tumor volume was observed at day 11 in the treated group. This effect was sustained through the end of the treatment, while no sign of toxicity was observed throughout the treatment period as depicted by a stable bodyweight (**Fig. 6D**). To determine if the growth inhibition was correlated to reduced cell proliferation and resistance to apoptosis, we sought to examine markers of tumor cell proliferation (Ki-67) and apoptosis (cleaved caspase-3) in PC3 tumor tissues treated with or without SD-208 by immunohistochemistry (IHC). As shown in **Fig. 6E-F**, IHC staining of cleaved caspase-3 showed increased apoptosis in tumor explants of the treated group as compared with the control. In accordance, a significant reduction in Ki-67 positive cells was observed in SD-208-treated groups of tumors, indicating inhibition of tumor cell proliferation by SD-208. Proteins such as survivin and Bcl-xL have been found to be associated with tumor survival, chemoresistance, and radioresistance [37] and have been described as biomarkers for PKD [15]. SD-208 treatment led to a moderate decrease in the protein expression level of these proteins in the treatment group compared to the control group (**Fig. 6G**). Also, decreased levels of p-S^473^-Akt and Akt were observed in SD-208 treated group as compared to the control, which is in concurrence of our previous study where PKD3 was shown to modulate Akt activity [10]. These effects of SD-208 on Bcl-xL, survivin, Akt, and p-Akt levels may, in part, account for its pro-apoptotic and antitumor activity in PC3 xenograft tumors. To demonstrate that SD-208 inhibited the intended target—PKD, we also determined the effect of SD-208 on PKD activity in tumor xenograft tissues. PKD2 was immunoprecipitated and subjected to *in vitro* PKD kinase assay. As shown in **Fig. 6H**, treatment with SD-208 significantly suppressed PKD2 activity in treated- vs. untreated-tumors, suggesting that SD-208 has reached the intended target and the inhibition of PKD may account for growth reduction of tumor xenografts.

## Discussion

Dysregulation of protein kinase D (PKD) contributes to sustained cell proliferation in multiple cellular systems including cancer [10,38,39]. Increasing evidence support the potential of PKDs as therapeutic target to combat various types of cancers including prostate cancer. Although several PKD small molecular inhibitors have been reported in the past, none have progressed to the clinic. This is mainly due to the lack of *in vivo* activity and sufficient therapeutic efficacy in these inhibitory chemotypes. To date, CRT0066101 remains the only selective PKD inhibitor that exhibits *in vivo* activity [15], clearly showing the need for more diverse, structurally distinct, and *in vivo*-active leads for further drug development. Here, we describe the *in vitro* and *in vivo* inhibition of PKD-mediated prostate tumor growth by a novel chemical inhibitor of PKD, SD-208.

In exploring putative binding modes of SD-208, we employed molecular modeling to determine key interactions associated with PKD inhibition. As a Type I ATP-competitive inhibitor, we anticipated SD-208 to exhibit classical hydrogen bond donor and acceptor interactions with residues in the hinge region [27,40]. Our modeling efforts indicated such hydrogen bond formation between SD-208’s pteridine core and the amino acid backbone of Leu^662^. Another feature of inhibition is described by a potentially critical contact between the nitrogen of the 4-aminopyridine and the charged side chain of Lys^612^. Primary sequence alignment indicates that Lys^612^ in PKD1 is a critical conserved residue required for ATP binding [41]. Previously reported modeling studies from Novartis suggested key contacts between their two PKD inhibitors and the charged side chain of Lys^612^ [18]. Their model positions a hydrogen bond accepter near Lys^612^ and their SAR analysis confirms this chemical feature as a critical component for inhibition. Our model of SD-208 locates the nitrogen of 4-aminopyridine near Lys^612^ where it can act as a hydrogen bond acceptor. Additionally, the SAR analysis further supports the notion that this nitrogen atom is important for PKD inhibition. Lastly, the phenyl ring of SD-208 is found in a conserved hydrophobic region near the entrance of the ATP binding site. Traditionally, this region is largely solvent exposed and can be exploited by both polar and hydrophobic groups [42]. Our PKD homology model presents the disubstituted phenyl ring between the hydrophobic amino acids Leu^589^ and Leu^713^, which may dictate the tolerance of aromatic substitutions. Therefore, our molecular modeling of SD-208 provided insights into the mechanism of inhibition and suggested key amino acid interactions associated with SD-208 binding.

SD-208 inhibited the growth of PC3 prostate cancer cells with an IC_50_ of 17μM, and its anti-proliferative activity was reversed by overexpression of PKD1 and PKD3, indicating inhibition of PKD accounts for the anti-proliferative effect of SD-208. This finding agrees our previous report that knockdown of PKD3, a predominantly expressed PKD isoform in PC3 cells, causes dramatic arrest in cell proliferation [10]. Additionally, we have shown for the first time that SD-208 caused systemic inhibition of tumor growth *in vitro* and *in vivo* in a prostate tumor xenograft model through inhibition of PKD, suggesting that this agent could potentially be exploited as a therapeutic agent for prostate cancer treatment. In this study, we have also demonstrated that SD-208 significantly blocked prostate cancer cell invasion. However, this effect may be primarily mediated through the inhibition of transforming growth factor β receptor I (TGF-βRI) since SD-208 has previously been reported as a TGF-βRI inhibitor [19] and several studies have shown that SD-208 blocked tumor invasion and metastasis by targeting TGF-βRI [19,43]. Other than TGF-βRI, inhibition of PKD may also account in part for this effect since our previous data have shown that inactivation of PKD retarded prostate cancer cell migration/invasion [22,24].

In the present study, we also demonstrated a novel molecular mechanism through which SD-208 inhibited prostate cancer cell proliferation. Our data showed that SD-208 exerted a strong anti-proliferative effect against prostate cancer cells by inducing G2/M arrest. The eukaryotic cell cycle progression is regulated by cyclin-dependent kinases (Cdks) and Cyclins. Progression through G1 into S phase is regulated by the Cyclin A-Cdk2 and Cyclin E-Cdk2 complexes, while Cyclin B-Cdc2 plays a role in G2/M phase transition [34]. Cip/p21 is a universal inhibitor of CDKs that also regulates the G2/M phase transitions by binding to CDK-Cyclin complex and inhibits its kinase activity [35]. Cdc25C is another critical regulator of Cdc2-Cyclin B1 kinase activity and control cell cycle progression by dephosphorylating and activating CDKs. Consistent with these notions, we observed an increase in the protein level of inhibitory p21 in both the cells lines. An increased phosphorylation of Cdc2 accompanied by a decreased Cdc25C protein and phosphorylation was also observed in both cell lines treated with SD-208. The increased Cdc2 phosphorylation was likely due to reduced Cdc25C protein level which prevented it from dephosphorylating Cdc2, thereby resulting in enhanced Cdc2 phosphorylation. Taken together, the induction of p21 at early stage, along with the increased p-Cdc2 as a result of the downregulation of Cdc25c at late stage, lies at the base of the mechanism through which SD-208 induced G2/M arrest.

In conclusion, this study identified SD-208 as a novel, potent, and *in vivo* active small molecule PKD inhibitor. We showed that this agent was orally bioavailable and blocked prostate cancer cells proliferation *in vitro* and *in vivo*. A series of analogs of SD-208 established the first PKD-related SAR of this lead structure and determined that an electron-deficient aromatic ring is preferred in zone 1, and that the 4-aminopyridine in zone 2 is essential for activity. For the first time, our study elucidated a unique molecular mechanism of action of SD-208 through induction of p21 along with increased phosphorylation of Cdc2 and Cdc25C, which contributed to G2/M arrest in prostate cancer cell proliferation. Our computational analyses provided additional insights into the possible molecular interactions of SD-208 with PKD and would help in optimizing SD-208 and its congeners for enhanced therapeutic efficacy as potential anticancer agents.

## Supporting Information

S1 Fig General synthetic scheme for the preparation of SD-208 analogs. (TIF)

S2 Fig PKD mediated Hsp27 activity in prostate cancer cells was inhibited by SD-208. LNCaP cells were pretreated with different doses of inhibitors for 45 min, followed by PMA stimulation at 10 nM for 20 min. Cell lysates were subjected to immunoblotting for p-S^910^-PKD1 and p-S^738/742^-PKD1. GAPDH was blotted as loading control. The experiment was repeated two times and the representative blots are shown.
(TIF)

S3 Fig PKD mediated Hsp27 activity in prostate cancer cells was inhibited by SD-208. PC3 cells were pretreated with different doses of inhibitors for 45 min, followed by PMA stimulation at 10 nM for 20 min. Cell lysates were subjected to immunoblotting for p-S^910^-PKD1 and p-S^738/742^-PKD1. GAPDH was blotted as loading control. The experiment was repeated two times and the representative blots are shown.
(TIF)

S1 File The Synthesis of SD-208 Inhibitors of Protein Kinase D. Experimental details and spectroscopic data for SD-208 analogs.
(DOC)

S2 File Supplemental Information on Methods. (DOCX)
