# Supplementary figures and images for: SD-208, a Novel Protein Kinase D Inhibitor, Blocks Prostate Cancer Cell Proliferation and Tumor Growth In Vivo by Inducing G2/M Cell Cycle Arrest
Source: PLoS One. 2015 Mar 6;10(3):e0119346. doi: 10.1371/journal.pone.0119346 (PMC4352033; doi:10.1371/journal.pone.0119346)

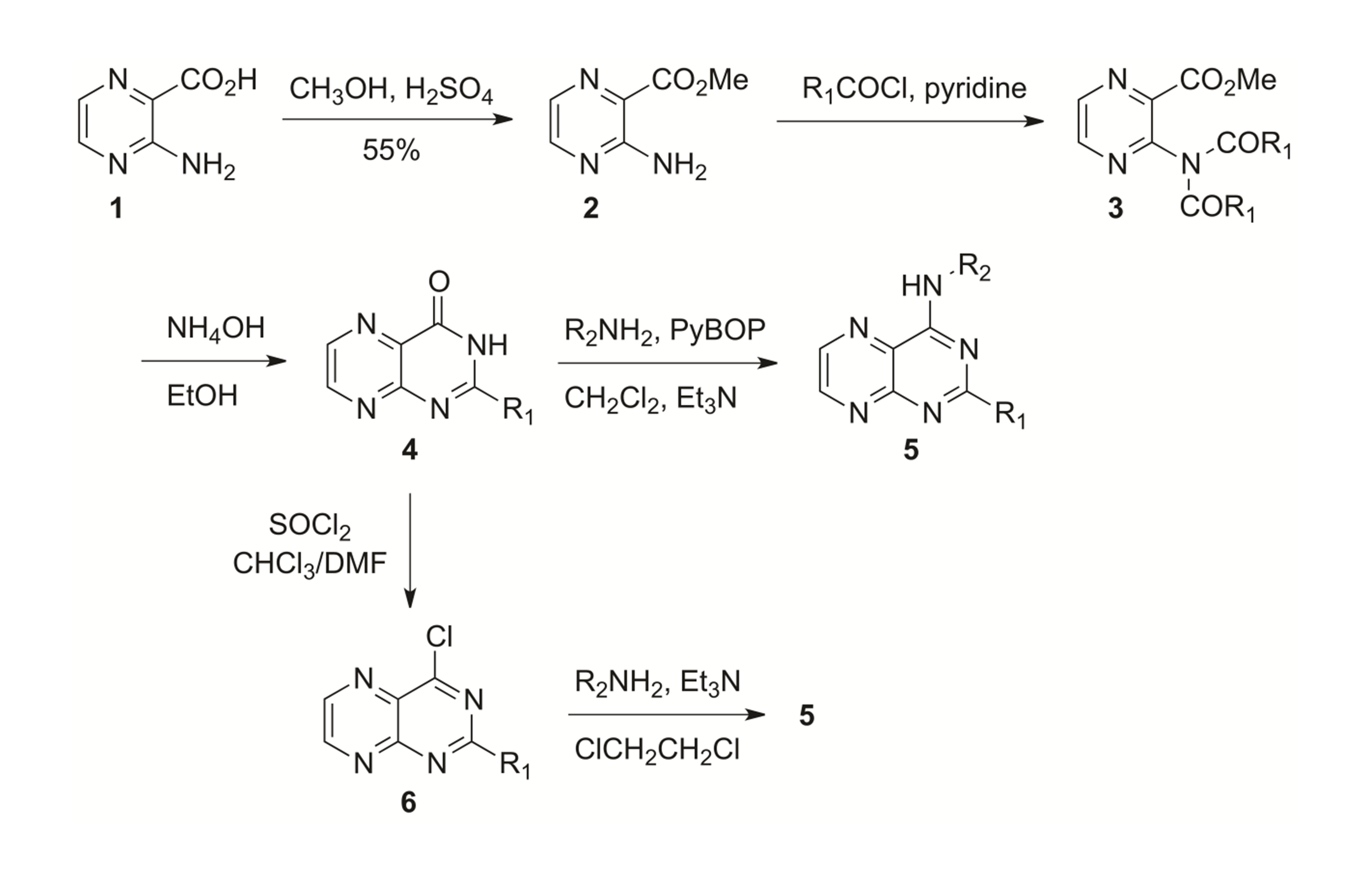

Supplement: S1 Fig — (TIF) [file pone.0119346.s001.tif]

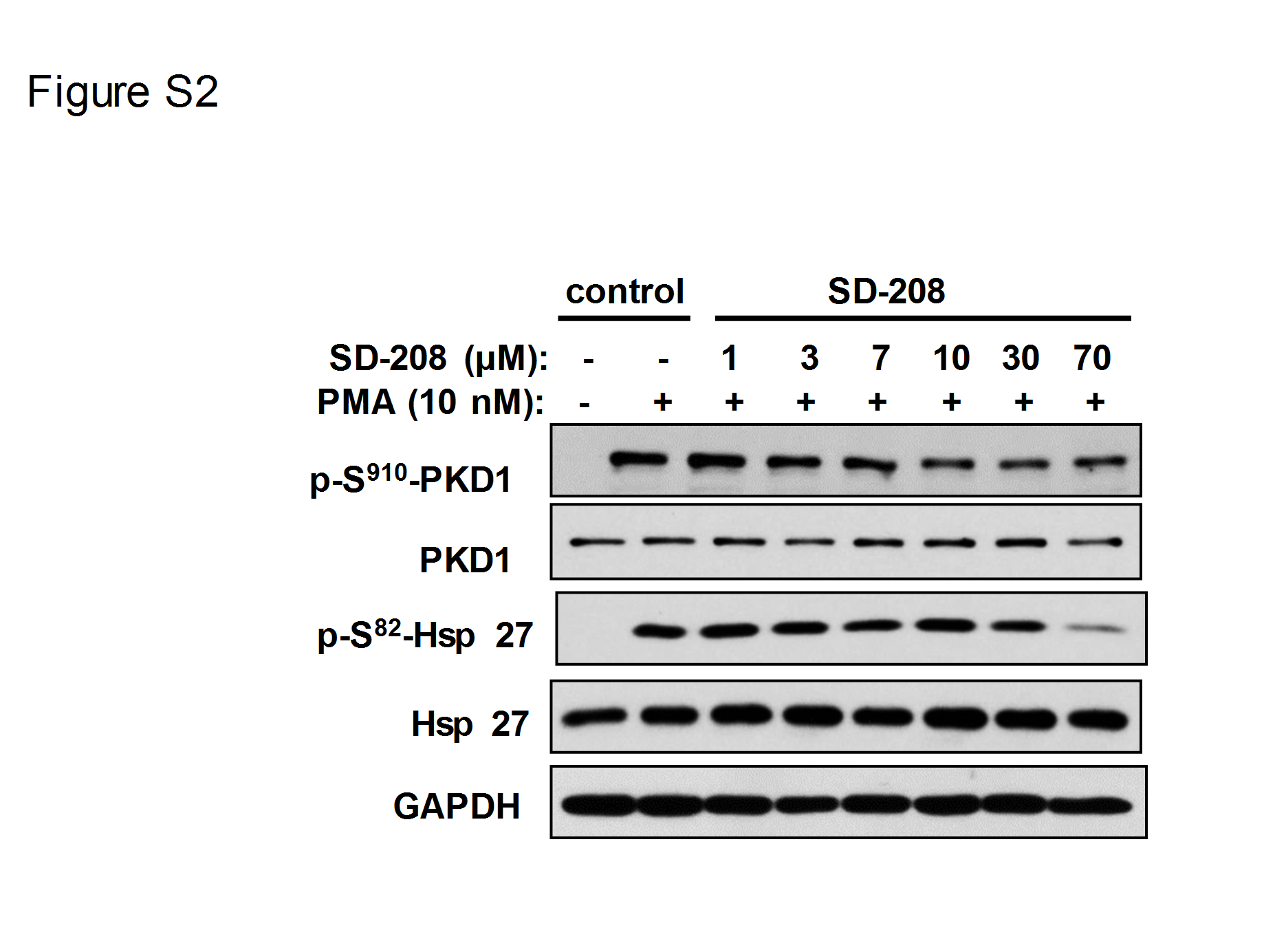

Supplement: S2 Fig — LNCaP cells were pretreated with different doses of inhibitors for 45 min, followed by PMA stimulation at 10 nM for 20 min. Cell lysates were subjected to immunoblotting for p-S910-PKD1 and p-S738/742-PKD1. GAPDH was blotted as loading control. The experiment was repeated two times and the representative blots are shown. (TIF) [file pone.0119346.s002.tif]

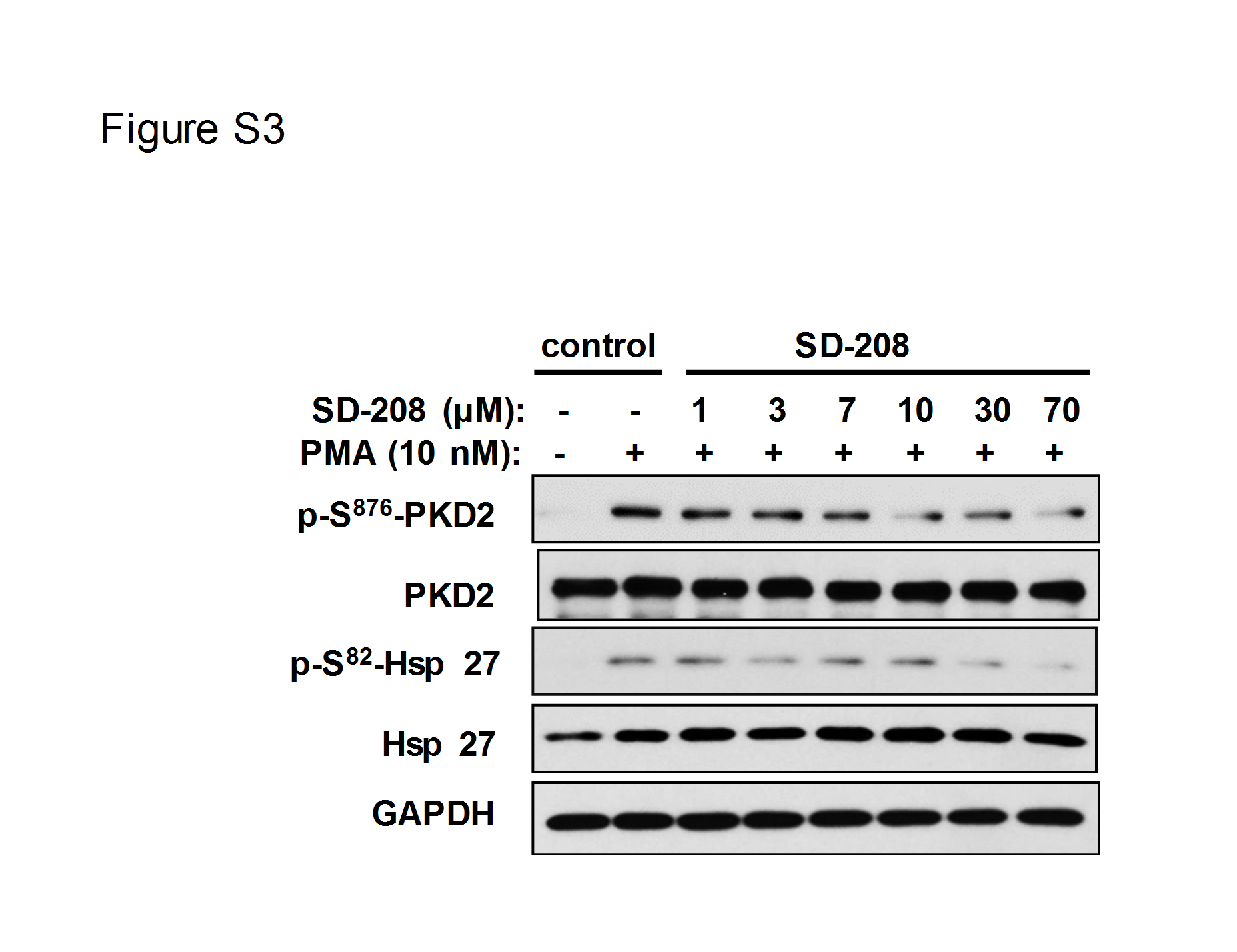

Supplement: S3 Fig — PC3 cells were pretreated with different doses of inhibitors for 45 min, followed by PMA stimulation at 10 nM for 20 min. Cell lysates were subjected to immunoblotting for p-S910-PKD1 and p-S738/742-PKD1. GAPDH was blotted as loading control. The experiment was repeated two times and the representative blots are shown. (TIF) [file pone.0119346.s003.tif]
