# Supplementary material for: SD-208, a Novel Protein Kinase D Inhibitor, Blocks Prostate Cancer Cell Proliferation and Tumor Growth In Vivo by Inducing G2/M Cell Cycle Arrest
Source: PLoS One. 2015 Mar 6;10(3):e0119346. doi: 10.1371/journal.pone.0119346 (PMC4352033; doi:10.1371/journal.pone.0119346)
Supplement: S1 File — Experimental details and spectroscopic data for SD-208 analogs. (DOC) [file pone.0119346.s004.doc]

**Supplementary Information**

**The Synthesis of SD-208 Inhibitors of Protein Kinase D**

**Manuj Tandon^1^, Joseph Salamoun^2^, Evan Carder^1^, Elisa Farber^2^, Shuping Xu^1^, Fan Deng^3^, Hua Tang^4^, Peter Wipf^2^, and Q. Jane Wang^1^**

**Table of Contents**

| **General Experimental** | S2 |
| --- | --- |
| **Representative Procedures** | S3 |
| 2-(5-Chloro-2-fluorophenyl)-*N*-(pyridine-4-yl)pteridin-4-amine **(SD-208)** | S3 |
| Methyl 3-aminopyrazine-2-carboxylate **(2)** | S4 |
| Methyl 3-(*N*-(5-chloro-2-fluorobenzoyl)-5-chloro-2-fluorobenzamido)pyrazine-2-carboxylate **(3j)** | S4 |
| 2-(5-Chloro-2-fluorophenyl)pteridin-4(3*H*)-one **(4j)** | S5 |
| 2,*N*-Diphenylpteridin-4-amine **(5a)** | S6 |
| Methyl 3-(*N*-benzoylbenzamido)pyrazine-2-carboxylate **(3a)** | S6 |
| Methyl 3-(*N*-(2,5-difluorobenzoyl)-2,5-difluorobenzamido)pyrazine-2-carboxylate **(3e)** | S7 |
| Methyl 3-(*N*-(trifluoromethylbenzoyl)-3-trifluoromethylbenzamido)pyrazine-2-carboxylate **(3h)** | S7 |
| Methyl 3-(*N*-(3,5-dichlorobenzoyl)-3,5-dichlorobenzamido)pyrazine-2-carboxylate **(3i)** | S8 |
| 2-Phenylpteridin-4(3*H*)-one **(4a)** | S8 |
| 2-(2,5-Difluorophenyl)pteridin-4(3*H*)-one **(4e)** | S9 |
| 2-(3-(Trifluoromethyl)phenyl)pteridin-4(3*H*)-one **(4h)** | S9 |
| 2-(2,5-Dichlorophenyl)pteridin-4(3*H*)-one **(4i)** | S10 |
| 2-Phenyl-*N*-(cyclohexyl)pteridin-4-amine **(5b)** | S10 |
| 2-Phenyl-*N*-(pyridin-4-yl)pteridin-4-amine **(5c)** | S11 |
| 2-Phenyl-*N*-(pyridin-3-yl)pteridin-4-amine **(5d)** | S11 |
| 2-(2,5-Difluorophenyl)-*N*-(pyridin-4-yl)pteridin-4-amine **(5e)** | S12 |
| 2-(2,5-Difluorophenyl)-*N*-(pyridin-3-yl)pteridin-4-amine **(5f)** | S12 |
| 2-(2,5-Difluorophenyl)-*N*-(2-morpholinoethyl)pteridin-4-amine **(5g)** | S13 |
| 2-(3-(Trifluoromethyl)phenyl)-*N*-(pyridin-4-yl)pteridin-4-amine **(5h)** | S13 |
| 2-(3,5-Dichlorophenyl)-*N*-(pyridin-4-yl)pteridin-4-amine **(5i)** | S14 |
| 2-(5-Chloro-2-fluorophenyl)-4-(piperazin-1-yl)pteridine **(5j)** | S14 |
| **Supplementary References** | S15 |

**General Experimental**. All air-sensitive reactions were performed under an N_2_ or Ar atmosphere. Reactions carried out at temperatures above room temperature (rt) employed an oil bath. EtOH was stored over 4 Å molecular sieves. Pyridine, CH_2_Cl_2_, and CHCl_3_ were distilled from CaH_2_. Et_3_N was stored over KOH. All commercial reagents were used as received. Concentrating under reduced pressure refers to removing solvents by the use of a rotary evaporator connected to a PIAB Lab Vac H40.

Reactions were monitored by thin layer chromatography analysis (EMD, pre-coated silica gel 60 F254 plates, 250 µm layer thickness) and visualization was accomplished with a 254 nm or 365 nm UV light and by staining with a phosphomolybdic acid solution (5.00 g of phosphomolybdic acid in 100 mL of 95% EtOH), *p*-anisaldehyde solution (2.50 mL of *p*-anisaldehyde, 2 mL of AcOH, and 3.5 mL of concentrated H_2_SO_4_ in 100 mL of 95% EtOH), KMnO_4_ solution (1.50 g of KMnO_4_ and 1.50 g of K_2_CO_3_ in 100 mL of a 0.1% NaOH solution), or Vaughn’s reagent (4.80 g of (NH_4_)_6_Mo_7_O_24_•4 H_2_O and 0.20 g of Ce(SO_4_)_2_ in 100 mL of a 3.5 M H_2_SO_4_) when needed. Flash chromatography on SiO_2_ (Silicycle, Silia-P Flash, or SiliaFlash® P60; 40-63 μm) was used to purify the crude reaction mixtures where indicated. All products were placed under high vacuum (0.5 – 4 mmHg) to remove trace solvents.

Melting points (Mp) were determined using a Laboratory Devices Mel-Temp II in open capillary tubes and are uncorrected. Infrared spectra (IR) were obtained from neat solids or oils on a Smiths Detection IdentifyIR FT-IR spectrometer. High-resolution mass spectra (HRMS) were obtained on a Micromass UK Limited, Q-TOF Ultima API or a Thermo Scientific Exactive Orbitrap LC-MS. Nuclear magnetic resonance (NMR) spectra were obtained on a Bruker Avance at 300 MHz, 400 MHz, 500 MHz, or 700 MHz in CDCl_3_ or (CD_3_)_2_SO. Chemical shifts (δ) were reported in parts per million with the residual solvent peak used as an internal standard δ ^1^H / ^13^C (solvent): 7.26 / 77.16 (CDCl_3_); 2.50 / 39.52 ((CD_3_)_2_SO). ^1^H NMR spectra were obtained and are tabulated as follows: chemical shift, multiplicity (s = singlet, d = doublet, dd = doublet of doublets, t = triplet, q = quartet, m = multiplet, bs = broad singlet), number of protons, and coupling constant(s). ^13^C NMR spectra were recorded using a proton-decoupled pulse sequence run at 100 MHz or 125 MHz and are tabulated as follows: chemical shift, multiplicity (d = doublet, dd = doublet of doublets, q = quartet), and coupling constant(s). All final compounds were at >95% purity based on NMR or liquid chromatography-mass spectrometry (LC-MS), unless stated otherwise.

**Representative Procedures**

**SD-208**

**2-(5-Chloro-2-fluorophenyl)-*N*-(pyridine-4-yl)pteridin-4-amine** **(SD-208)** (1)**.** Et_3_N (0.29 mL, 2.0 mmol) was added to a solution of **4j** (0.20 g, 0.65 mmol), 4-aminopyridine (0.13 g, 1.3 mmol), and PyBOP (0.68 g, 1.3 mmol) in CH_2_Cl_2_ (5 mL) at rt under N_2_. The suspension was stirred for 24 h and filtered through SiO_2_ plug (10% MeOH/EtOAc). The filtrate was concentrated under reduced pressure. The residue was stirred in hot H_2_O (50 mL) for 30 min, filtered, and washed with rt H_2_O (100 mL) to yield **SD-208** (0.092 g, 40%) as a brown solid: Mp 223-227 °C; IR (ATR) 1577, 1340, 1171, 816 cm^-1^; ^1^H NMR ((CD_3_)_2_SO, 400 MHz) δ 11.16 (s, 1 H), 9.29 (d, 1 H, *J =* 1.6 Hz), 9.06 (d, 1 H, *J =* 1.6 Hz), 8.58 (d, 2 H, *J* = 5.2 Hz), 8.31 (d, 2 H, *J =* 5.2 Hz), 8.21 (dd, 1 H, *J* = 6.4, 2.4 Hz), 7.71-7.68 (m, 1 H), 7.50 (app t, 1 H, *J* = 10.4 Hz); ^13^C NMR ((CD_3_)_2_SO, 125 MHz) δ 160.0 (d, *J_CF_* = 3.8 Hz), 159.5 (d, *J_CF_* = 255.3 Hz), 158.9, 153.7, 152.5, 148.8, 146.6, 145.2, 132.4 (d, *J_CF_* = 8.8 Hz), 131.3, 128.4 (d, *J_CF_* = 2.5 Hz), 127.5 (d, *J_CF_* = 10.1 Hz), 125.2, 119.3 (d, *J_CF_* = 23.9 Hz), 115.2; HRMS (ESI^+^) *m/z* calculated (calcd) for C_17_H_11_ClFN_6_ (M+H)^+^ 353.0712, found 353.0709.

**2**

**Methyl 3-aminopyrazine-2-carboxylate** **(2)** (2, 3)**.** Concentrated H_2_SO_4_ (10 mL) was added slowly to a suspension of 3-aminopyrazine-2-carboxylic acid (**1**) (3.27 g, 23.5 mmol) in MeOH (30 mL) at 0 °C. The resulting mixture was stirred for 2 d at rt. The suspension turned to a dark red solution. The solution was cooled to 0 °C and basified with an aqueous solution of NaHCO_3_ to pH 8. The precipitate was filtered and recrystallized from hot methanol (25 mL) to yield **2** (1.98 g, 55%) as an orange solid: ^1^H NMR (CDCl_3_, 500 MHz) δ 8.21 (d, 1 H, *J* = 2.0 Hz), 8.00 (d, 1 H, *J* = 2.0 Hz), 3.99 (s, 3 H); ^13^C NMR (CDCl_3_, 125 MHz) δ 167.0, 156.1, 147.7, 133.8, 124.5, 52.9.

.

**3j**

**Methyl 3-(*N*-(5-chloro-2-fluorobenzoyl)-5-chloro-2-fluorobenzamido)pyrazine-2-carboxylate (3j)** (4)**.** 5-Chloro-2-fluorobenzoyl chloride (1.79 mL, 13.1 mmol) was added dropwise to a solution of **2** (1.00 g, 6.53 mmol) in pyridine (13 mL) and the resulting orange mixture was stirred at 50 ºC under N_2_ for 2.5 h. The mixture was allowed to cool to rt and quenched with NaHCO_3_. The solvent was removed under reduced pressure and the residue was passed through a short SiO_2_ column (CH_2_Cl_2_ then EtOAc) to yield a crude yellow oil. The oil was triturated with MeOH (30 mL) under sonication resulting in a precipitate that was filtered to yield **3j** (2.51 g, 82%) as a white solid: Mp 141-144 °C; IR (ATR) 3027, 1724, 1694, 1484, 1398, 1232, 1215 cm^-1^; ^1^H NMR (CDCl_3_, 300 MHz) δ 8.64 (d, 1 H, *J =* 2.4 Hz), 8.49 (d, 1 H, *J =* 2.4 Hz), 7.77 (dd, 2 H, *J =* 6.0, 2.7 Hz), 7.44-7.37 (m, 2 H), 7.02-6.91 (m, 2 H), 3.98 (s, 3 H); ^13^C NMR (CDCl_3_, 100 MHz) δ 166.5, 163.3, 158.1 (d, *J_CF_* = 255.5 Hz), 147.9, 145.8, 143.3, 140.5, 134.2 (d, *J_CF_* = 8 Hz), 130.9, 130.0, 124.1 (d, *J_CF_* = 13.7 Hz), 117.9 (d, *J_CF_* = 23.9 Hz), 53.7; HRMS (ESI^+^) *m/z* calcd for C_20_H_11_Cl_2_F_2_N_3_O_4_Na (M+Na)^+^ 487.9992, found 488.0003.

**4j**

**2-(5-Chloro-2-fluorophenyl)pteridin-4(3*H*)-one** **(4j)** (4)**.** NH_4_OH (30 mL, 28% solution) was added slowly in hourly aliquots to a suspension of **3j** (0.950 g, 2.04 mmol) in EtOH (10 mL) while the reaction mixture was stirred under reflux until reaction completion. The solvent was removed under reduced pressure and the residue was washed with a hot solution of hexanes/EtOAc (1:1, 60 mL) to yield **4j** (0.436 g, 77%) as a light brown solid: Mp >150 °C (dec.); IR (ATR) 3027, 1707, 1601, 1480, 1392, 1036, 815 cm^-1^; ^1^H NMR ((CD_3_)_2_SO, 300 MHz) δ 13.21 (bs, 1 H), 9.04 (d, 1 H, *J =* 2.1 Hz), 8.88 (d, 1 H, *J =* 2.1 Hz), 7.88 (dd, 1 H, *J* = 6.0, 2.4 Hz), 7.77-7.71 (m, 1 H), 7.50 (app t, 1 H, *J* = 9.9 Hz); ^13^C NMR ((CD_3_)_2_SO, 100 MHz) δ 161.7, 158.3 (d, *J_CF_* = 252.5 Hz), 154.9, 153.3, 150.2, 144.5, 133.3, 132.8 (d, *J_CF_* = 8.8 Hz), 130.7, 128.3 (d, *J_CF_* = 3.0 Hz), 123.9 (d, *J_CF_* = 14.6 Hz), 118.4 (d, *J_CF_* = 23.6 Hz); HRMS (ESI^+^) m/z calcd for C_12_H_7_ClFN_4_O (M+H)^+^ 277.0287, found 277.0281.

**5a**

**2,*N*-Diphenylpteridin-4-amine** **(5a)**. Thionyl chloride (0.08 mL, 1 mmol) was added to a solution of **4a** (0.050 g, 0.22 mmol) in CHCl_3_ (1.8 mL) and DMF (35 μL) and the resulting solution was stirred under reflux for 50 min. The volatiles were removed under reduced pressure and the crude product was triturated with Et_2_O to give a green solid. The solid (0.030 g) was dissolved in 1,2-dichloroethane (1 mL) and to the solution was added freshly distilled aniline (0.03 mL, 0.3 mmol) and then Et_3_N (0.06 mL, 0.4 mmol) dropwise. The resulting solution was stirred at 70 °C for 4 h. The solvent was removed under reduced pressure and the crude product was purified by chromatography on SiO_2_ (hexanes/EtOAc, 65:45) and then triturated with Et_2_O (10 mL) to yield **5a** (0.020 g, 40% (>90% purity by LC-MS)) as a yellow solid: Mp 160 °C (dec.); IR (ATR) 3355, 3066, 2936, 1599, 1581, 1562, 1407, 1329 cm^-1^; ^1^H NMR (CDCl_3_, 400 MHz) δ 9.09 (d, 1 H, *J* = 1.6 Hz), 9.09-9.07 (m, 1 H), 8.71 (d, 1 H, *J* = 2.0 Hz), 8.70-8.68 (m, 2 H), 8.05 (d, 2 H, *J* = 7.6 Hz), 7.56-7.50 (m, 5 H), 7.25-7.23 (m, 1 H); ^13^C NMR (CDCl_3_, 125 MHz) δ 165.1, 158.2, 154.6, 151.3, 142.7, 138.1, 137.7, 131.6, 129.5, 129.4, 128.6, 125.8, 124.6, 120.7; HRMS (ESI^+^) *m/z* calcd for C_18_H_14_N_5_ (M+H)^+^ 300.1249, found 300.1242.

**3a**

**Methyl 3-(*N*-benzoylbenzamido)pyrazine-2-carboxylate (3a)** (5)**.** According to the procedure used for **3j**, benzoyl chloride (4.55 mL, 39.2 mmol) and **2** (1.20 g, 7.84 mmol) were converted to **3a** (2.05 g, 72%): Mp 189-192 °C; IR (ATR) 1733, 1696, 1402, 1312, 1238, 1113 cm^-1^; ^1^H NMR (CDCl_3_, 500 MHz) δ 8.53 (d, 1 H, *J* = 2.5 Hz), 8.41 (d, 1 H, *J* = 2.0 Hz), 7.85 (d, 4 H, *J* = 7.5 Hz), 7.48 (t, 2 H, *J* = 7.5 Hz), 7.37 (t, 4 H, *J* = 8.0 Hz), 3.94 (s, 3 H); ^13^C NMR (CDCl_3_, 125 MHz) δ 172.9, 164.2, 149.7, 145.8, 142.0, 140.0, 134.5, 132.9, 129.4, 128.9, 53.6; HRMS (ESI^+^) *m/z* calcd for C_20_H_15_N_3_ Na O_4_ (M+Na)^+^ 384.0960, found 384.0959.

**3e**

**Methyl 3-(*N*-(2,5-difluorobenzoyl)-2,5-difluorobenzamido)pyrazine-2-carboxylate (3e)**. According to the procedure used for **3j**, 2,5-difluorobenzoyl chloride (4.03 mL, 32.7 mmol) and **2** (1.00 g, 6.53 mmol) were converted to **3e** (1.62 g, 57%): Mp 118-120 °C; IR (ATR) 3081, 1728, 1679, 1487, 1415, 1264, 1212 cm^-1^; ^1^H NMR (CDCl_3_, 500 MHz) δ 8.62 (d, 1 H, *J* = 2.5 Hz), 8.47 (d, 1 H, *J* = 2.5 Hz), 7.52-7.48 (m, 2 H), 7.17-7.11 (m, 2 H), 7.01-6.96 (m, 2 H), 3.97 (s, 3 H); ^13^C NMR (CDCl_3_, 125 MHz) δ 166.6, 163.4, 158.4 (dd, *J_CF_* = 246.9, 3.3 Hz), 155.8 (dd, *J_CF_* = 254.8, 4.9 Hz), 147.9, 145.8, 143.2, 140.4, 123.8 (dd, *J_CF_* = 14.2, 7.9 Hz), 121.1 (dd, *J_CF_* = 23.4, 9.3 Hz), 117.9 (dd, *J_CF_* = 24.6, 8.7 Hz), 117.5 (d, *J* = 26.8 Hz), 53.7; HRMS (ESI^+^) *m/z* calcd for C_20_H_12_F_4_N_3_O_4_ (M+H)^+^ 434.0764, found 434.0762.

**3h**

**Methyl 3-(*N*-(trifluoromethylbenzoyl)-3-trifluoromethylbenzamido)pyrazine-2-carboxylate (3h)** (4)**.** According to the procedure used for **3j**, 3-trifluoromethylbenzoyl chloride (2.46 mL, 16.3 mmol) and **2** (0.50 g, 3.3 mmol) were converted to **3h** (0.92 g, 56%): Mp 118-120 °C; IR (ATR) 1700, 1404, 1335, 1253, 1131, 1118 cm^-1^; ^1^H NMR (CDCl_3_, 400 MHz) δ 8.62 (d, 1 H, *J* = 2.0 Hz), 8.47 (d, 1 H, *J* = 2.0 Hz), 8.09 (s, 2 H), 7.99 (d, 2 H, *J* = 7.6 Hz), 7.74 (d, 2 H, *J* = 7.6 Hz), 7.52 (t, 2 H, *J* = 7.6 Hz), 3.98 (s, 3 H); ^13^C NMR (CDCl_3_, 100 MHz) δ 171.4, 164.1, 149.1, 146.1, 142.8, 140.1, 135.2, 132.1, 131.7 (q, *J_CF_* = 33.1 Hz), 129.6, 129.5 (q, *J_CF_* = 3.5 Hz), 126.4 (q, *J_CF_* = 3.8 Hz), 123.4 (q, *J_CF_* = 272.6 Hz), 53.9; HRMS (ESI^+^) *m/z* calcd for C_22_H_14_F_6_N_3_O_4_ (M+H)^+^ 498.0889, found 498.0869.

**3i**

**Methyl 3-(*N*-(3,5-dichlorobenzoyl)-3,5-dichlorobenzamido)pyrazine-2-carboxylate (3i)**. According to the procedure used for **3j**, 3,5-dichlorobenzoyl chloride (3.42 g, 16.3 mmol) and **2** (0.50 g, 3.3 mmol) were converted to **3i** (1.03 g, 61%): Mp 190 °C (dec.); IR (ATR) 3083, 3066, 1719, 1698, 1566, 1400, 1275, 1230, 1212 cm^-1^; ^1^H NMR (CDCl_3_, 400 MHz) δ 8.64 (d, 1 H, *J* = 2.0 Hz), 8.50 (d, 1 H, *J* = 2.0 Hz), 7.65 (d, 4 H, *J =* 1.6 Hz), 7.48 (d, 2 H, *J* = 1.6 Hz), 4.00 (s, 3 H); ^13^C NMR (CDCl_3_, 100 MHz) δ 170.1, 164.1, 148.6, 146.2, 143.1, 139.9, 136.9, 135.9, 132.9, 127.4, 54.0; HRMS (ESI^+^) *m/z* calcd for C_20_H_12_Cl_4_N_3_O_4_ (M+H)^+^ 497.9582, found 497.9550.

**4a**

**2-Phenylpteridin-4(3*H*)-one (4a)** (6)**.** According to the procedure used for **4j**, **3a** (0.50 g, 1.4 mmol) was converted to **4a** (0.23 g, 74%): Mp >185 °C (dec.); IR (ATR) 3124, 1674, 1597, 1540, 1476, 1320, 1130 cm^-1^; ^1^H NMR (CDCl_3_, 300 MHz) δ 10.83 (bs, 1 H), 9.01 (d, 1 H, *J* = 1.5 Hz), 8.84 (d, 1 H, *J* = 1.5 Hz), 8.26 (d, 2 H, *J* = 6.3 Hz), 7.67-7.59 (m, 3 H); ^13^C NMR ((CD)_3_SO, 125 MHz) δ 161.5, 155.9, 155.0, 150.4, 144.1, 133.1, 132.2, 132.0, 128.7, 128.3; HRMS (ESI^+^) *m/z* calcd for C_12_H_9_N_4_O (M+H)^+^ 225.0776, found 225.0773.

**4e**

**2-(2,5-Difluorophenyl)pteridin-4(3*H*)-one** **(4e)**. According to the procedure used for **4j**, **3e** (0.38 g, 0.89 mmol) was converted to **4e** (0.12 g, 50%): Mp >163 °C (dec.); IR (ATR) 3081, 1704, 1605, 1582, 1458, 1396, 1182 cm^-1^; ^1^H NMR ((CD_3_)_2_SO, 500 MHz) δ 13.22 (bs, 1 H), 9.04 (d, 1 H, *J* = 1.5 Hz), 8.88 (s, 1 H), 7.69-7.65 (m, 1 H), 7.58-7.48 (m, 2 H); ^13^C NMR (CDCl_3_, 125 MHz) δ 160.8, 158.7, 155.8 (dd, *J_CF_* = 243.8, 5.9 Hz), 152.5, 150.5, 144.9, 133.4, 122.8 (dd, *J_CF_* = 15.8, 8.5 Hz), 120.1 (dd, *J_CF_* = 24.0, 8.8 Hz), 118.3 (dd, *J_CF_* = 24.1, 8.6 Hz), 117.5 (d, *J_CF_* = 26.0 Hz); HRMS (ESI^+^) *m/z* calcd for C_12_H_7_F_2_N_4_O (M+H)^+^ 261.0588, found 261.0577.

**4h**

**2-(3-(Trifluoromethyl)phenyl)pteridin-4(3*H*)-one (4h)** (4)**.** According to the procedure used for **4j**, **3h** (0.80 g, 1.6 mmol) was converted to **4h** (0.25 g, 53%): Mp >225 °C (dec.); IR (ATR) 3081, 2867, 1711, 1579, 1540, 1327, 1266, 1111 cm^-1^; ^1^H NMR ((CD_3_)_2_SO, 500 MHz) 13.31 (s, 1 H), 9.04 (d, 1 H, *J* = 2.0 Hz), 8.86 (d, 1 H, *J* = 2.0 Hz), 8.57 (s, 1 H), 8.52 (d, 1 H, *J* = 8.0 Hz), 8.02 (d, 1 H, *J* = 8.0 Hz), 7.85 (t, 1 H, *J* = 7.5 Hz); ^13^C NMR ((CD_3_)_2_SO, 125 MHz) δ 161.5, 154.8, 154.6, 150.5, 144.5, 133.4, 133.1, 132.3, 130.1, 129.5 (q, *J_CF_* = 32.4 Hz), 128.6 (q, *J_CF_* = 3.6 Hz), 125.0 (q, *J_CF_* = 3.1 Hz), 123.9 (q, *J_CF_* = 273.4 Hz); HRMS (ESI^-^) *m/z* calcd for C_13_H_6_F_3_N_4_O (M-H)^-^ 291.0494, found 291.0529.

**4i**

**2-(2,5-Dichlorophenyl)pteridin-4(3*H*)-one (4i)**. According to the procedure used for **4j**, **3i** (0.80 g, 1.6 mmol) was converted to **4i** (0.26 g, 54% (>90% purity by ^1^H NMR)): Mp >255 °C (dec.); IR (ATR) 3079, 1713, 1586, 1558, 1540, 1398, 1292 cm^-1^; ^1^H NMR ((CD_3_)_2_SO, 500 MHz) δ 13.20 (bs, 1 H), 9.01 (bs, 1 H), 8.83 (bs, 1 H), 8.24 (bs, 2 H), 7.90 (bs, 1 H); ^13^C NMR ((CD_3_)_2_SO, 125 MHz) δ 161.7, 154.7, 153.9, 150.4, 144.5, 135.7, 134.5, 133.4, 131.3, 127.0; HRMS (ESI^+^) *m/z* calcd for C_12_H_7_Cl_2_N_4_O (M+H)^+^ 292.9997, found 292.9983.

**5b**

**2-Phenyl-*N*-(cyclohexyl)pteridin-4-amine** **(5b)** (6)**.** According to the procedure used for **5a**, **4a** (0.070 g, 0.31 mmol) and *c*-hexylamine (0.05 mL, 0.5 mmol) were converted to **5b** (0.020 g, 21%) as an oil: IR (ATR) 3390, 2926, 2852, 1568, 1402, 1333 cm^-1^; ^1^H NMR (CDCl_3_, 500 MHz) δ 8.97 (d, 1 H, *J* = 2.0 Hz), 8.68-8.64 (m, 2 H), 8.56 (d, 1 H, *J* = 2.0 Hz), 7.51-7.48 (m, 3 H), 7.03 (d, 1 H, *J* = 7.5 Hz), 4.41-4.34 (m, 1 H), 2.22-2.19 (m, 2 H), 1.86 (dt, 2 H, *J* = 13.5, 3.5 Hz), 1.73 (dt, 2 H, *J* = 13.5, 3.5 Hz), 1.59-1.50 (m, 2 H), 1.48-1.41 (m, 1 H), 1.36-1.25 (m, 1 H); ^13^C NMR (CDCl_3_, 125 MHz) δ 164.9, 160.0, 154.6, 150.8, 141.9, 138.0, 131.2, 129.3, 128.4, 125.9, 50.1, 32.8, 25.8, 25.0; HRMS (ESI^+^) *m/z* calcd for C_18_H_20_N_5_ (M+H)^+^ 306.1719, found 306.1724.

**5c**

**2-Phenyl-*N*-(pyridin-4-yl)pteridin-4-amine** **(5c)**. According to the procedure used for **SD-208**, **4a** (0.050 g, 0.22 mmol) and 4-aminopyridine (0.042 g, 0.44 mmol) were converted to **5c** (0.050 g, 71%): Mp >250 °C (dec.); IR (ATR) 3437, 3217, 1657, 1592, 1543, 1448, 1366, 1146 cm^-1^; ^1^H NMR ((CD_3_)_2_SO, 400 MHz) δ 9.46 (s, 1 H), 9.30 (bs, 1 H), 9.21 (s, 1 H), 9.01 (d, 2 H, *J* = 7.2 Hz), 8.65 (d, 2 H, *J* = 6.4 Hz), 7.69-7.65 (m, 3 H), 7.18 (d, 2 H, *J* = 6.4 Hz); ^13^C NMR ((CD_3_)_2_SO, 100 MHz) δ 161.9, 160.8, 158.4, 156.3, 153.9, 147.5, 142.8, 135.4, 132.6, 129.2, 128.9, 127.4, 109.0; HRMS (ESI^+^) *m/z* calcd for C_17_H_13_N_6_ 301.1202, found 301.1196.

**5d**

**2-Phenyl-*N*-(pyridin-3-yl)pteridin-4-amine (5d)**. According to the procedure used for **SD-208**, **4a** (0.070 g, 0.31 mmol) and 3-aminopyridine (0.059 g, 0.62 mmol) were converted to **5d** (0.064 g, 65%): Mp >265 °C (dec.); IR (ATR) 3061, 1596, 1562, 1534, 1463, 1428, 1335, 1185, 1089 cm^-1^; ^1^H NMR ((CD_3_)_2_SO/CD_2_Cl_2_, 9:1, 500 MHz) δ 9.38 (d, 1 H, *J* = 1.5 Hz), 9.23 (d, 1 H, *J* = 1.5 Hz), 8.64-8.61 (m, 2 H), 8.59 (d, 1 H, *J* = 8.0 Hz), 8.15 (d, 1 H, *J* = 8.0 Hz), 8.02 (t, 1 H, *J* = 7.5 Hz), 7.74 (t, 1 H, *J* = 7.5 Hz), 7.70-7.67 (m, 3 H); HRMS (ESI^+^) *m/z* calcd for C_17_H_13_N_6_ (M+H)^+^ 301.1196, found 301.1193.

**5e**

**2-(2,5-Difluorophenyl)-*N*-(pyridin-4-yl)pteridin-4-amine (5e)**. According to the procedure used for **SD-208**, **4e** (0.050 g, 0.19 mmol) and 4-aminopyridine (0.036 g, 0.38 mmol) were converted to **5e** (0.003 g, <5%): IR (ATR) 3353, 2947, 1705, 1605, 1577, 1549, 1394 cm^-1^; ^1^H NMR (CDCl_3_, 500 MHz) δ 9.19 (d, 2 H, *J* = 1.5 Hz), 8.83 (d, 1 H, *J* = 1.5 Hz), 8.66 (d, 2 H, *J* = 5.5 Hz), 8.15-8.11 (m, 1 H), 8.02 (dd, 2 H, *J* = 5.0, 1.5 Hz), 7.25-7.21 (m, 2 H); ^13^C NMR (CDCl_3_, 125 MHz) δ 162.3 (dd, *J_CF_* = 3.8, 1.3 Hz), 158.7 (dd, *J_CF_* = 241.5, 2.5 Hz), 158.4, 158.1 (dd, *J_CF_* = 252.8, 2.5 Hz), 154.2, 152.2, 151.0, 144.7, 144.2, 127.2 (dd, *J_CF_* = 10.2, 8.0 Hz), 125.0, 119.5 (dd, *J_CF_* = 24.2, 9.1 Hz), 118.9, 118.8 (dd. *J* = 25.2, 2.5 Hz), 118.6 (dd, *J_CF_* = 25.2, 8.8 Hz); HRMS (ESI^+^) *m/z* calcd for C_17_H_11_F_2_N_6_ (M+H)^+^ 337.1013, found 337.1014.

**5f**

**2-(2,5-Difluorophenyl)-*N*-(pyridin-3-yl)pteridin-4-amine** **(5f)**. According to the procedure used for **SD-208**, **4e** (0.10 g, 0.38 mmol) and 3-aminopyridine (0.073 g, 0.77 mmol) were converted to **5f** (0.070 g, 51%): Mp >255 °C (dec.); IR (ATR) 3381, 2971, 1535, 1430, 1344 cm^-1^; ^1^H NMR ((CD_3_)_2_SO, 700 MHz) δ 9.42 (s, 1 H), 9.32 (s, 1 H), 8.63 (d, 1 H, *J* = 8.4 Hz), 8.15 (d, 1 H, *J* = 8.4 Hz), 8.09-8.06 (m, 1 H), 7.99 (t, 1 H, *J* = 7.7 Hz), 7.28 (t, 1 H, *J* = 7.7 Hz), 7.62-7.58 (m, 2 H); ^13^C NMR ((CD_3_)_2_SO, 176 MHz) δ characteristic signals 156.0, 154.5, 153.3, 148.1, 133.8, 132.6, 131.3, 127.0, 126.5, 115.2; HRMS (ESI^+^) *m/z* calcd for C_17_H_11_F_2_N_6_ (M+H)^+^ 337.1013, found 337.1021.

**5g**

**2-(2,5-Difluorophenyl)-*N*-(2-morpholinoethyl)pteridin-4-amine** **(5g)**. According to the procedure used for **SD-208**, **4e** (0.10 g, 0.38 mmol) and 2-morpholinoethanamine (0.10 mL, 0.77 mmol) were converted to **5g** (0.078 g, 52%) as an oil: IR (ATR) 2859, 2244, 1584, 1568, 1495, 1445 cm^-1^; ^1^H NMR (CDCl_3_, 400 MHz) δ 9.02 (d, 1 H, *J* = 1.6 Hz), 8.67 (d, 1 H, *J* = 2.0 Hz), 8.03-7.98 (m, 1 H), 7.68 (bs, 1 H), 7.16-7.10 (m, 2 H), 3.82 (dd, 2 H, *J* = 11.2, 5.6 Hz), 3.78-3.76 (m, 4 H), 2.78-2.72 (m, 2 H), 2.62-2.57 (m, 4 H); ^13^C NMR (CDCl_3_, 100 MHz) δ 162.8 (dd, *J_CF_* = 2.0 Hz), 160.8, 158.5 (dd, *J_CF_* = 241.6, 2.4 Hz), 157.9 (dd, *J_CF_* = 255.8, 2.2 Hz), 154.1, 151.1, 143.2, 128.1 (dd, *J_CF_* = 10.7, 7.6 Hz), 125.6, 118.7-118.0 (m, 3 C), 67.1, 56.8, 53.5, 37.5; HRMS (ESI^+^) *m/z* calcd for C_18_H_19_F_2_N_6_O (M+H)^+^ 373.1588, found 373.1590.

**5h**

**2-(3-(Trifluoromethyl)phenyl)-*N*-(pyridin-4-yl)pteridin-4-amine** **(5h)** (4)**.** According to the procedure used for **SD-208**, **4h** (0.10 g, 0.34 mmol) and 4-aminopyridine (0.064 g, 0.68 mmol) were converted to **5h** (0.030 g, 23%): Mp >165 °C (dec.); IR (ATR) 3062, 1599, 1575, 1545, 1512, 1439, 1338 cm^-1^; ^1^H NMR (CDCl_3_, 500 MHz) δ 9.16 (s, 1 H), 9.14 (s, 1 H), 8.93 (s, 1 H), 8.79 (d, 1 H, *J* = 8.0 Hz), 8.77 (s, 1 H), 8.68 (bs, 2 H), 7.94 (d, 2 H, *J* = 5.0 Hz), 7.80 (d, 1 H, *J* = 7.5 Hz), 7.67 (t, 1 H, *J* = 8.0 Hz); ^13^C NMR (CDCl_3_, 125 MHz) δ 163.3, 158.5, 154.4 152.1, 151.1, 144.7, 143.8, 138.0, 132.4, 131.4 (q, *J_CF_* = 32.6 Hz), 129.3, 128.3 (q, *J_CF_* = 3.5 Hz), 126.3 (q, *J_CF_* = 4.0 Hz), 125.4, 124.2 (q, *J_CF_* = 272.3 Hz), 114.4; HRMS (ESI^+^) *m/z* calcd for C_18_H_12_F_3_N_6_ (M+H)^+^ 369.1070, found 369.1064.

**5i**

**2-(3,5-Dichlorophenyl)-*N*-(pyridin-4-yl)pteridin-4-amine** **(5i)**. According to the procedure used for **SD-208**, **4i** (0.070 g, 0.24 mmol) and 4-aminopyridine (0.045 g, 0.48 mmol) were converted to **5i** (0.004 g, <5%) as an oil: IR (ATR) 3342, 2924, 2850, 1601, 1579, 1551, 1525, 1445 cm^-1^; ^1^H NMR (CDCl_3_, 400 MHz) δ 9.29 (bs, 1 H), 9.19 (d, 1 H, *J* = 1.6 Hz), 8.82 (d, 1 H, *J* = 2.0 Hz), 8.73 (d, 2 H, *J* = 6.0 Hz), 8.53 (d, 2 H, *J* = 1.6 Hz), 8.03 (d, 2 H, *J* = 6.0 Hz), 7.55 (t, 1 H, *J* = 1.6 Hz); ^13^C NMR (CDCl_3_, 125 MHz) δ characteristic signals 162.4, 158.5, 154.4, 152.4, 144.2, 140.1, 135.7, 131.7, 129.1, 127.8, 125.4, 114.6; HRMS (ESI+) *m/z* calcd for C_17_H_11_Cl_2_N_6_ (M+H)^+^ 369.0422, found 369.0449.

**5j**

**2-(5-Chloro-2-fluorophenyl)-4-(piperazin-1-yl)pteridine** **(5j).** According to the procedure used for **SD-208**, **4j** (0.10 g, 0.36 mmol) and piperazine (0.16 g, 1.8 mmol) were converted to **5j** (0.016 mg, 13%): Mp 173-177 °C; IR (ATR) 3286, 3059, 2919, 1547, 1506, 1433, 1333, 811 cm^-1^; ^1^H NMR ((CD_3_)_2_SO, 300 MHz): δ 9.05 (d, 1 H, *J* = 1.8 Hz), 8.82 (d, 1 H, *J =* 1.8 Hz), 8.12 (dd, 1 H, *J* = 6.6, 3.0 Hz), 7.67-7.60 (m, 1 H), 7.41 (dd, 1 H, *J* = 10.5, 9.3 Hz), 4.38 (bs, 4 H), 2.89 (t, 4 H, *J* = 5.1 Hz); ^13^C NMR ((CD_3_)_2_SO, 100 MHz): δ 159.5 (d, *J_CF_* = 255.2 Hz), 159.4 (d, *J_CF_* = 5.1 Hz), 159.1, 155.5, 150.3, 141.7, 131.7 (d, *J_CF_* = 9.1 Hz), 131.0, 128.1 (d, *J_CF_* = 9.0 Hz), 126.9, 119.2, 118.9, 46.0; HRMS (ESI^+^) m/z calcd for C_16_H_15_ClFN_6_ (M+H)^+^ 345.1025, found 345.1023.

**Supplementary References**

1. Raboisson PJMB, Surleraux DLN, Lin TI, Lenz O, Simmen KA, inventors; Tibotec Pharmaceuticals Ltd., assignee. Pteridines useful as HCV Inhibitors and Methods for the Preparation Thereof. United States patent US 20090156595A1. 2009 Jun. 18.

2. Ellingson RC, Henry RL, McDonald FG. Pyrazine Chemistry. I. Derivatives of 3-Aminopyrazinoic Acid. J Am Chem Soc. 1945;67:1711-3.

3. Duan H, Ning M, Chen X, Zou Q, Zhang L, Feng Y, et al. Design, Synthesis, and Antidiabetic Activity of 4-Phenoxynicotinamide and 4-Phenoxypyrimidine-5-carboxamide Derivatives as Potent and Orally Efficacious TGR5 Agonists. J Med Chem. 2012;55:10475-89.

4. Raboisson P, Lenz O, Lin T-I, Surleraux D, Chakravarty S, Scholliers A, et al. Evaluation of the anti-hepatitis C virus effect of novel potent, selective, and orally bioavailable JNK and VEGFR kinase inhibitors. Bioorg Med Chem Lett. 2007;17:1843-9.

5. Wamhoff H, Kroth E. Dihalogentriphenylphosphorane in der Heterocyclensynthese, 29. Eine einfache Synthese von Pteridin-4-onen aus 3-Amino-2-pyrazincarbonsäuremethylester und Pyrazino[3,1]oxazin-4-onen. Synthesis. 1994:405-10.

6. Giorgi I, Biagi G, Livi O, Leonardi M, Scartoni V, Pietra D. Synthesis of New 2-Phenyladenines and 2-Phenylpteridines and Biological Evaluation as Adenosine Receptor Ligands. Archiv der Pharmazie. 2007;340:81-7.
