# Supplementary material for: SD-208, a Novel Protein Kinase D Inhibitor, Blocks Prostate Cancer Cell Proliferation and Tumor Growth In Vivo by Inducing G2/M Cell Cycle Arrest
Source: PLoS One. 2015 Mar 6;10(3):e0119346. doi: 10.1371/journal.pone.0119346 (PMC4352033; doi:10.1371/journal.pone.0119346)
Supplement: S2 File — (DOCX) [file pone.0119346.s005.docx]

**SD-208, a Novel Protein Kinase D Inhibitor, Blocks Prostate Cancer Cell Proliferation and Tumor Growth *In Vivo* by Inducing G2/M Cell Cycle Arrest**

**Manuj Tandon^1^, Joseph Salamoun^2^, Evan J. Carder^1^, Elisa Farber^2^, Shuping Xu^1^, Fan Deng^3^, Hua Tang^4^, Peter Wipf^2^, and Q. Jane Wang^1^**

From the ^1^Department of Pharmacology and Chemical Biology, and ^2^Department of Chemistry, University of Pittsburgh, Pittsburgh, PA 15260, and the ^3^Department of Cell Biology, School of Basic Medical Sciences, Southern Medical University, Guangzhou, China, and the ^4^Department of Cellular and Molecular Biology, University of Texas Health Science Center at Tyler, Tyler, Texas 75708.

**Corresponding Author**: Q. Jane Wang, Ph.D., Department of Pharmacology and Chemical Biology, University of Pittsburgh School of Medicine, E1354 BST**,** Pittsburgh, PA 15261. Telephone: 412-383-7754; Fax: 412-648-1945; Email: [qjw1@pitt.edu](mailto:qjw1@pitt.edu)

**Supplement File S2**

**Supplemental Information on Methods**

**Synthesis of SD-208 analogs**

Fischer esterification of commercially available 3-aminopyrazine-2-carboxylic acid (**1**) with concentrated sulfuric acid in methanol gave the amino ester **2** in 55% yield (**Supplement Figure S1**) ([31](#_ENREF_31)). Bis-acylation of the aromatic amine **2** with the desired benzoyl chloride furnished imide **3**, establishing the first point of variability (*zone 1*) ([32](#_ENREF_32)). Then, bis-acylated intermediate **3** was subjected to an ammonium hydroxide mediated cyclization to pteridinone **4**, completing the construction of the pteridine core ([32](#_ENREF_32)). The second point of variability (*zone 2*) was introduced by amination of pteridinone **4** with selected amines in the presence of the peptide coupling reagent, benzotriazol-1-yl-oxytripyrrolidinophosphonium hexafluorophosphate (PyBOP), to provide analogue **5** ([32](#_ENREF_32), [33](#_ENREF_33)). Alternatively, analogue **5** can be prepared by chlorination of the carbonyl moiety in **4** with thionyl chloride under reflux conditions to give the chlorinated intermediate **6** followed by nucleophilic aromatic substitution with the desired amine ([32](#_ENREF_32)) Table 1 provides an overview of synthesized compounds and yields for individual steps.

***In Vitro* Radiometric PKD Inhibitor Screening Assay**

1.2 µM of a HDAC5 peptide was used as substrate in the reaction. Phosphorylation of HDAC5 peptide was detected in a kinase reaction having 1 µCi [γ-^32^P] ATP (Perkin Elmer Life Sciences), 25 µM ATP, 50 ng purified recombinant PKD1 in 50 µL kinase buffer containing 50 mM Tris-HCl, pH 7.5, 4 mM MgCl_2_ and 10 mM β-mercaptoethanol. The reaction was incubated at 30 ^o^C for 10 min and 25 μL of the reaction was spotted on Whatman P81 filter paper. The filter paper was washed 3 times in 0.5% phosphoric acid, air dried, and counted using a Beckman LS6500 multipurpose scintillation counter. The percent PKD1 inhibition was graphed using GraphPad Prism software 5.0.

**Wound Healing Assay**

PC3 cells were grown to confluence in 6-well plates and treated with 0.5 µM Mitomycin C for 2 hrs. The monolayer was “wounded” by scraping the monolayer with a pipette tip. The indicated concentration of compound was added to the media, and the wound was imaged immediately under an inverted phase-contrast microscope with 200X magnification. After 24 h, a final image was taken. The wound gap was measured, and % wound healing was calculated as the % wound healing (wound gap at 0 h - wound gap at 24 h)/wound gap at 0 h x 100.The average % wound healing was determined based on at least 6 measurements of the wound gap.

**Matrigel Invasion Assay**

DU145 cells (8.0 × 10^4^ cells/mL) were seeded into the top chamber of BioCoat control inserts (pore size 8 µM) or BioCoat Matrigel invasion inserts with Matrigel-coated filters (BD Biosciences, Bedford, MA). To stimulate invasion, medium in the lower chamber of the insert contained 20% FBS. Inhibitors were added at 30 μM to both the upper and lower chambers, and cells were incubated at 37 ^o^C for 22 h. After incubation, non-invasive cells in the top chamber were removed using a cotton swab, and invasive cells that have penetrated the filters were fixed in 100% methanol and stained with 0.4% hematoxylin. After staining, cells were counted under a microscope (200 x magnification). The percent invasion was expressed as the number of cells that migrated through the Matrigel-coated chamber relative to the number of cells that migrated through the control insert in at least 5 random fields.
